# Integrity of the circadian clock determines regularity of high-frequency and diurnal LFP rhythms within and between brain areas

**DOI:** 10.1038/s41380-024-02795-z

**Published:** 2024-10-29

**Authors:** Paul Volkmann, Annika E. I. Geiger, Anisja Hühne-Landgraf, Nina Miljanovic, Jessica Bly, Tobias Engl, Heidrun Potschka, Moritz J. Rossner, Dominic Landgraf

**Affiliations:** 1 Circadian Biology Group, Section of Molecular Neurobiology, Department of Psychiatry and Psychotherapy, LMU University Hospital, 80336 Munich, Germany; 2 Molecular Neurobiology Group, Department of Psychiatry and Psychotherapy, LMU University Hospital, 80336 Munich, Germany; 3 Institute of Pharmacology, Toxicology, and Pharmacy, LMU, 80539 Munich, Germany; 4 Systasy Bioscience GmbH, 81669 Munich, Germany; 5 Present Address: Centre for Neural Circuits and Behaviour, University of Oxford, OX1 3SR, Oxford, UK

**Keywords:** Neuroscience, Addiction

## Abstract

Circadian clocks control most physiological processes of many species. We specifically wanted to investigate the influence of environmental and endogenous rhythms and their interplay on electrophysiological dynamics of neuronal populations. Therefore, we measured local field potential (LFP) time series in wild-type and *Cryptochrome 1* and *2* deficient (*Cry1/2*^*−/−*^) mice in the suprachiasmatic nucleus and the nucleus accumbens under regular light conditions and constant darkness. Using refined descriptive and statistical analyses, we systematically profiled LFP time series activity. We show that both environmental and endogenous rhythms strongly influence the rhythmicity of LFP signals and their frequency components, but also shape neuronal patterns on much smaller time scales, as neuronal activity in *Cry1/2*^*−/−*^ mice is significantly less regular but at each time more synchronous within and between brain areas than in wild-type mice. These results show that functional circadian rhythms are integral for both circadian and non-circadian coordination of neuronal ensemble dynamics.

## Introduction

Many medical conditions, most notably psychiatric disorders, are based on dysfunctions of the brain and its numerous areas and structures that are interconnected and collectively generate and control all behavior and multiple physiological processes [1]. Optimal functioning of processes within and communication between different brain regions requires temporal coordination [2]. This includes adaptation to the environment and its recurring changes over the 24-hour day to which an organism is exposed [3]. Organisms can react spontaneously to the changes in environmental conditions and adapt their behavior and bodily functions to them repeatedly. However, in order to anticipate the daily recurring changes and not just spontaneously tune behavior and body functions to them, many have evolved endogenous systems to generate autonomous 24-h oscillations that are synchronized to environmental rhythms - so-called circadian (from Latin *circa*, around, and *dies*, day) clocks [4–7]. Endogenous clocks increase or decrease the activity of virtually all physiological processes, depending on requirements that prevail during a given period of the day. Among others, these processes are known to include gene expression, metabolism, cardiovascular function, the immune system, neuronal activity, and behavior [8–10]. At a cellular level, rhythmicity emerges from the molecular interplay of so-called core clock genes and proteins that form a transcription-translation feedback loop, in short TTL [4]. Fundamental elements of this system in mammals are CLOCK and BMAL1 [11] that drive transcription of two other sets of genes, *Period 1*, *2*, and *3* (*Per1/2/3*) and *Cryptochrome 1* and *2* (*Cry1/2*) [12], yet more regulatory elements are involved. The mammalian hypothalamus is the site of the suprachiasmatic nucleus (SCN), which is considered the main pacemaker of the circadian system and the most important interface between rhythmic light signals from the environment and circadian clocks in the body [13]. The neuronal activity of the SCN is rhythmic and transmits its own rhythmicity to other tissues, including other brain regions, whose own neuronal activity in turn oscillates in a 24-h rhythm [14, 15].

The electrical activity of the brain can be observed at different micro-, meso-, and macroscales, e.g., by electrophysiological recordings on the single cell level or electroencephalograms able to cover different brain areas [16]. Previous studies have been able to identify an interplay between single neuron dynamics [17–21] or multi-unit activity [14, 22] and the circadian clock. In contrast, local field potentials (LFP), known to capture the summation of roughly 1,000 neurons in cortical recordings in the form of extracellular potential changes as ensemble activity [23, 24], have proven to be a valuable measure of activity levels within many different subregions. Circadian LFP oscillations have been reported to occur in the SCN [25, 26] as well as in the cerebellum and striatum [27].

Many brain areas harbor their own autonomous circadian clocks [28]. In addition, multiple structures of the brain receive direct or indirect signals from the retina about whether it is light or dark in the environment [29]. However, it is not known to what extent 24-h rhythms of electrical activity are driven either endogenously or exogenously, and to what extent rhythms from different brain regions, e.g., those of the SCN and subordinate brain regions, are interrelated [30]. Furthermore, it remains unclear whether endogenous circadian rhythms and exogenous light rhythms and their interplay, beyond the imposition of 24-h rhythms, influence integrity of high-frequency brain electrical activity. We hypothesize that these factors contribute to the interaction of neuronal signals between brain regions and suggest that the presence of endogenous and exogenous diurnal oscillations not only determines 24-h periodic overall activity levels, but also influences non-circadian patterns and the composition of neuronal activity within and between brain regions.

To approach these assumptions in a hypothesis testing study, we examined LFP signals with implanted telemetric small-sized electrodes from two brain regions of freely behaving mice with four combinations of different genetic and light conditions that decipher the respective influence of endogenous and exogenous rhythms and their interplay on electrical brain activity. As brain regions, we chose the SCN as the core pacemaker of the circadian system and the nucleus accumbens (NAc), a region with SCN-independent rhythmicity [28] and only indirect connections to the SCN [31]. To test the mere influence of endogenous clocks on electrical activity in these two regions, we compared LFP signals from wild-type and endogenously arrhythmic *Cry1/2*^*−/−*^ mice under constant darkness (DD). To examine exogenous influences, we subjected the same *Cry1/2*^*−/−*^ mice to 12:12 light-dark (LD) conditions. Finally, to investigate the interaction of endogenous and exogenous influences, we used the same wild-type mice in a 12:12 LD regimen as a fourth condition. Our results show that both endogenous and exogenous rhythms as well as their interplay influence the 24-h oscillation of concerted electrical activity in both brain regions and furthermore determine regularity and complexity of LFPs within and between them.

## Methods

### Ethics approval

All animal experiments were carried out in accordance with institutional guidelines and regulations approved by the Regierungspräsidium Oberbayern, ROB Munich, Germany under the license ROB-55.2-2532.Vet_02-16-179. All further study procedures were performed in accordance with institutional guidelines and safety regulations.

### Animals

*Cry1/2*^*−/−*^; *Per2*^*Luc*^ mice with C57BL/6 J background were kindly provided by Michael Hastings, MRC Laboratory of Molecular Biology, Cambridge, UK. Mice were backcrossed to C57BL/6 J background mice from our stock every 5 to 10 generations. Pairing of littermates was carried out to obtain two experimental groups of five male animals each: *Cry1/2*^*−/−*^; *Per2*^*Luc*^ (in this study referred to as *Cryptochrome 1* and *2* deficient / *Cry1/2*^*−/−*^) and *Cry1/2*^*+/+*^; *Per2*^*Luc*^ (in this study referred to as wild-type) mice. In total, seven animals per group ran through all experiments to finally get five of these animals per group (W1-W5 and C1-C5), whose recording data were used for statistical analyses. Two animals per group had to be excluded (see below). Surgery and recordings were conducted between the age of 8 to 14 weeks. Mice were group-housed (maximum of six animals) in ventilated cages (Tecniplast S.p.A, Italy) before and kept separated after surgery to avoid injuries. Food and water were provided ad libitum. Mice were maintained under a 12-hour light:12-hour dark cycle (LD), with lights being switched on at 7AM. After recording under LD conditions, mice were kept under constant darkness (DD). The assignment of animals was not randomized; experimenters were not blinded to animal’s genotype. Minimal number of animals necessary was determined by power analyses, aiming at an effect size > 2 (in order to primarily detect differences in fitted sine wave amplitudes), an intended power of 0.9, and an error probability of 0.05, yielding a required sample size of at least 5 animals.

### Implantation of transmitter and LFP probes

For recordings, we used the Data Sciences International (DSI, USA) telemetry system. The transmitter HD-X02 was prepared as described previously [32]. For creating electrodes, we used a single full hard PFA coated stainless steel wire (Science Products GmbH, Germany) with a diameter of 102 µm for both SCN and NAc [33, 34] and a calculated impedance around 150 kΩ. The transmitter implantation was carried out as described previously [35, 36]. Briefly, animals were allowed to habituate in the experimental room for 30 min before start of the surgery. Mice were anesthetized by injecting an intraperitoneal mix of Midazolam (5 mg/kg), Medetomidin (0.5 mg/kg), and Fentanyl (0.05 mg/kg). Depth of anesthesia was secured throughout surgery by checking the toe pinch reflex, body temperature, and respiratory rate. Eyes were covered with Dexpanthenol (Bepanthen, Bayer, Germany) to avoid dehydration. To expose the surgical field, the head was shaved down to the left side of the back. Mice were then fixed in a stereotactic frame (Stoelting, Germany). Negative leads were wrapped around a screw (slotted cheese head machine screws BN 650, Bossard, Germany) that was fixed on the skull above the cerebellum. Electrodes were inserted into the left SCN and the right NAc with leads attached. More specifically, they were implanted at −0.35 mm AP; 0.20 mm ML; −5.60 mm VD for SCN and +1.94 mm AP; −0.60 mm ML; −4.70 mm VD for the NAc in accordance with a reference atlas [37]. Paladur (Kulzer, Germany) was used to form an isolating coating around the electrodes, leads, and screw. The skin was then laid around the formed hat and closed with the appropriate amount of suture. To reverse anesthesia at the end of surgery, mice were injected with a mix of Atipamezole (2.5 mg/kg), Flumazenil (0.5 mg/kg), and Naloxone (1.2 mg/kg) intraperitoneally. Mice were also given Carprofen (5 mg/kg) subcutaneously on the first, second, and third postoperative day.

### LFP recordings

After surgery, animals were allowed proper time to recover (minimum of two weeks) in individual home cages. For recordings, mice were transferred to metabolic cages (TSE Systems, Germany) in which we also recorded locomotor activity by means of infrared beams with a 15-minute time resolution for one animal of each genotype (W5 and C5). The cages were placed on top of a receiver plate (DSI) catching the live signal of implanted transmitters. LFP recordings were obtained at a sampling rate of 500 Hz using Ponemah software (DSI). After 5 days of habituation, recordings under LD condition took place for three days. This was followed by a 5-day period of habituation to DD conditions and then recording under DD for three days.

### Imaging of brain slices

After deeply anesthetizing mice with 4% isoflurane and perfusion by intracardial injection of 1× phosphate-buffered saline and then 4% paraformaldehyde (PFA), the entire brain was removed and stored at 4 °C in 4% PFA for 24 h, then in a 15% sucrose solution for 24 h, afterwards in a 30% sucrose solution for three days or until it sank, and finally frozen at −80 °C. For imaging, brains were serially sliced at 40 µm using a cryostat microtome (Leica Biosystems, Germany). Brain slices were stained with hematoxylin-eosin. The correct location of probes was confirmed microscopically.

### Data processing

Raw LFP recordings were high-pass filtered at 0.5 Hz and visually inspected for artifacts, both with and without the help of the automatic artifact detection of the NeuroScore software (DSI). No animal displayed more than 30 artifacts in the analyzed time window, all artifacts exceeding the threshold of 1 mV were removed. Two wild-type and two *Cry1/2*^*−/−*^ animals were excluded on pre-established criteria after inspecting the raw LFP traces and displaying them as spectrogram before any further analyses took place as they showed flat LFP signals without distinct frequency content. Subsequent histological examination showed hemorrhage around the tip of the electrodes for the excluded animals which could explain the lack of signal, and which was not present in included animals.

Traces were then decomposed into their frequency components by fast Fourier transformation [38] using NeuroScore. More specifically, we obtained the spectral power of frequency bands for each delta (0.5–4 Hz), theta1 (4–6 Hz), theta2 (6–8 Hz), alpha1 (8–11 Hz), alpha2 (11–14 Hz), sigma (14–16 Hz), beta1 (16–18 Hz), beta2 (18–32 Hz), gamma1 (32–48 Hz), and gamma2 (48–70 Hz) within 1 s bins (for cross- and autocorrelation) or 4 s bins (for all other analyses of spectral data), respectively. We obtained data in 4-second bins as this provided a good compromise between frequency resolution of spectral power and time resolution but used 1-second bins for cross- and autocorrelation to capture their relevant dynamical time windows.

High-pass filtered raw LFP traces were used for a limited number of analyses: at their sampling rate of 500 Hz (for displaying raw LFP traces, continuous wavelet transforms, and coherence measurement), within 1-second bins (for cross- and autocorrelation), and within 4-second bins (for all other analyses).

After data was processed as described, the complete recording period was displayed as spectrogram (see below) and the first undisturbed and uninterrupted 24-hour period starting at 7AM corresponding for both brain regions was used for each light regimen for every individual animal for consecutive analyses (except for Lomb-Scargle analyses for which we used the entire length of each light regimen’s three consecutive recording days).

### Statistical analyses

All analyses of processed data presented in this study were carried out using R version 4.2.2 [39] and RStudio (RStudio, USA). Details of statistical tests are indicated in the figure legends and can be found in the Suppl. File. All analyses included the same n = 5 animals per genotype, all animals were included in every analysis. All outcome measures of our study are reported. Secondary measures describing properties of time series derived from our analyses (such as, for example, block count, sine wave amplitude, or correlation values – see below) which met normality (as determined by Q-Q plots) were compared using Type II ANOVA (or unpaired student’s t-test, respectively). When normality was not assumed (e.g., deviation of peaks from 24 h where data is necessarily right-skewed) or met (e.g., Euclidean distance), a general multivariate regression model (or Wilcoxon rank-sum test, respectively) was used. Sample size was identical for each group compared in every test. Therefore, variance of groups was not compared as the parametric tests used are robust against differences in variances in this case [40]. Differences were considered significant when p < 0.05.

Where single representative animals are shown, we always display the same wild-type (W1) and *Cry1/2*^*−/−*^ (C1) animal. The only exceptions are the raw LFP traces in Fig. S2A–C to show our recordings are not contaminated by locomotor activity as we only recorded metabolic cage activity data for animals W5 and C5, as well as the time-frequency plane in Fig. S3A. The experimenter was not blinded to the groups during data analysis.

#### Plotting

Most plots were created using the R function *ggplot* from the *ggplot2* package [41]. For spectrograms, LFP relative amplitude series, and matrix generation, the R function *pheatmap* from the *pheatmap* package [42] was used. Sine waves, cross-/autocorrelation, and continuous wavelet transform were plotted using the *plot* function of the *graphics* package.

#### LFP analyses

**Fig. 1:**
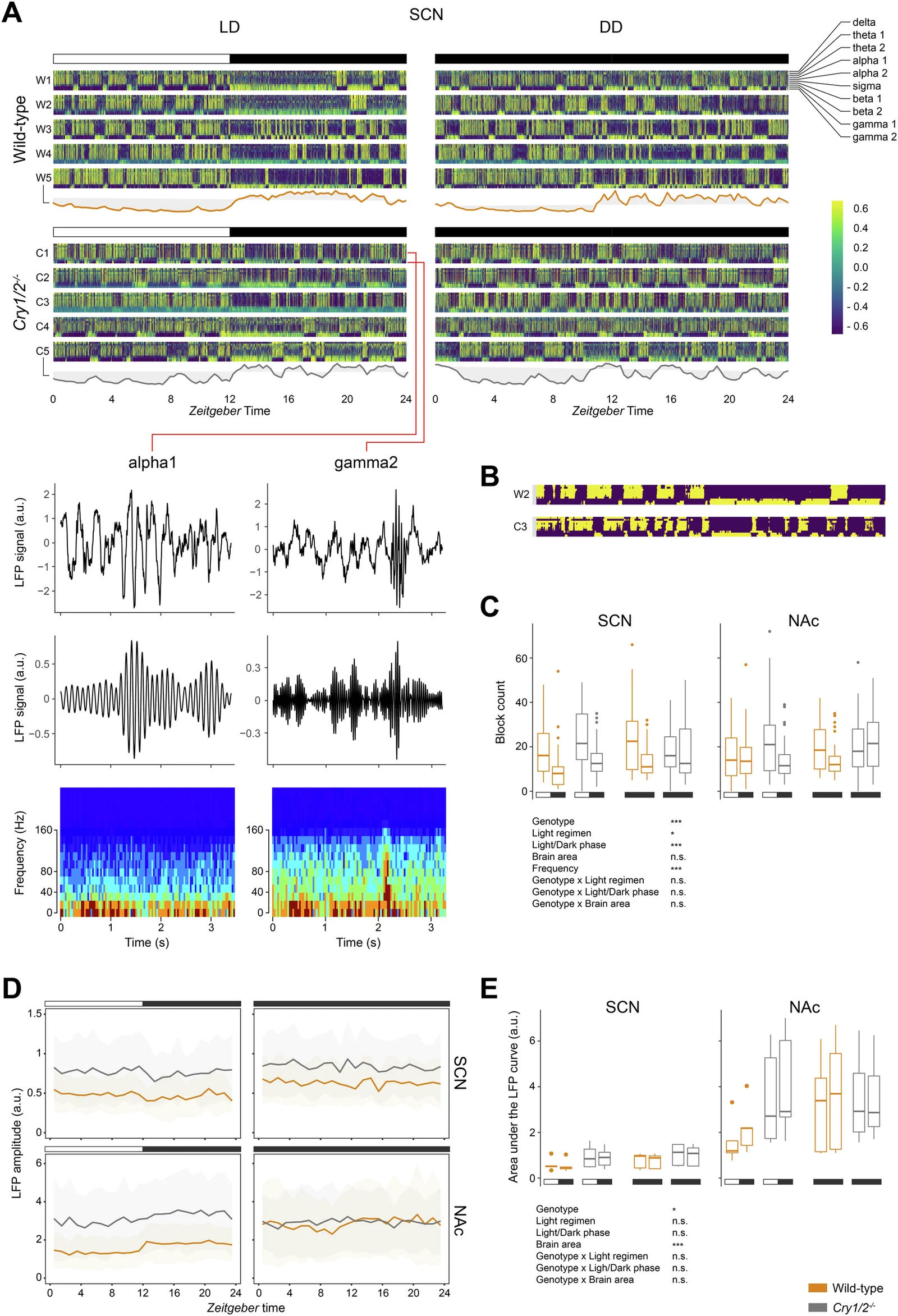
Disruptions of endogenous and environmental circadian rhythms increase fragmentation and intensity of LFP activity over 24 h. **A** Individual spectrograms of z-scored 24-hour SCN frequency activity of all animals. Color graded values refer to standard deviation. For W5 and C5, their corresponding locomotor activity levels are depicted. For C1, exemplary 3.6 s raw LFP traces under LD showing alpha1 (left, ZT18.1555) and gamma2 (right, ZT17.5065) oscillatory events in the SCN are displayed. The top trace is the raw signal, the middle trace the signal filtered at 8-11 Hz (alpha1) or 48-70 Hz (gamma2), respectively. The corresponding spectrograms are at the bottom. **B** Example 24-h block spectrograms (SCN under LD). Grey blocks at the beginning are a calculation artifact of the rolling average (the first 250 bins). **C** Comparison of number of blocks counted from block spectrograms. **D** 24-h time series of LFP amplitude with mean ± standard deviation within one-hour bins. **E** Area under the curve of the fast Fourier transformed LFP signal. Boxplots show median and whiskers indicating 1.5-fold distance of inter-quartile range (IQR) to the upper/lower quartile. White and black bars represent light and dark, respectively. Asterisks in (**C**) and (**E**) refer to Type II ANOVA. * p < 0.05, ** p < 0.01, *** p < 0.001; n.s. = not significant. a.u. = arbitrary unit.

Spectrograms for exemplary raw LFP traces shown in Fig. 1A, S2A–C, and S3C were created using R function *specgram* from the package *signal* [43] with a Fourier transform window size of 32.

The area under the curve of the fast Fourier transformed LFP signal in Fig. 1E was calculated by integrating over the absolute values of the transformed 4-second binned 24-hour time series of individual animals and pooling the respective results.

#### Continuous wavelet transform

We computed the continuous wavelet transform [44, 45] of raw LFP traces using Morlet wavelets with a width of 7 cycles [46] to obtain representative information about the occurrence of neural oscillatory events using the *wt* function of R package *biwavelet* [47] which we show in Fig. S3. This was carried out for every individual animal both during LD and DD in the SCN and NAc at ZT0, ZT6, ZT12, and ZT18 over the course of 6 min at each chosen *Zeitgeber* time. We then determined significance levels of the detected wavelet events and thresholded at a significance level of 1.96. We counted the occurrence of bouts above threshold for every individual period (frequency) and identified two frequencies – 66.8 and 8.3 Hz – centered around which we found local maxima for both genotypes. We show this quantification in Fig. S3A. We then calculated the percentiles of power within individual frequencies of analyzed traces and defined events as peaks that exceed the 98^th^ percentile in the time-frequency plane within their frequency [46]. From this data, we derived the frequency span of neural oscillatory events in the identified frequencies and displayed it in Fig. S3B as histogram. We then used filtered LFP traces (8–11 Hz for alpha1, 48–70 Hz for gamma2) to detect local maxima of neural oscillatory events for both regions in an example animal and identified times with high density of such maxima. We used this information to depict exemplary traces of neural oscillatory events in Fig. 1A and S3C. We further quantified event duration and interval and displayed them as density plots in Fig. S3D. We also quantified event power as fraction of the frequency specific median and again displayed them as density plots Fig. S3E based on peaks that exceed the 98^th^ percentile.

#### Z-scoring

**Fig. 2:**
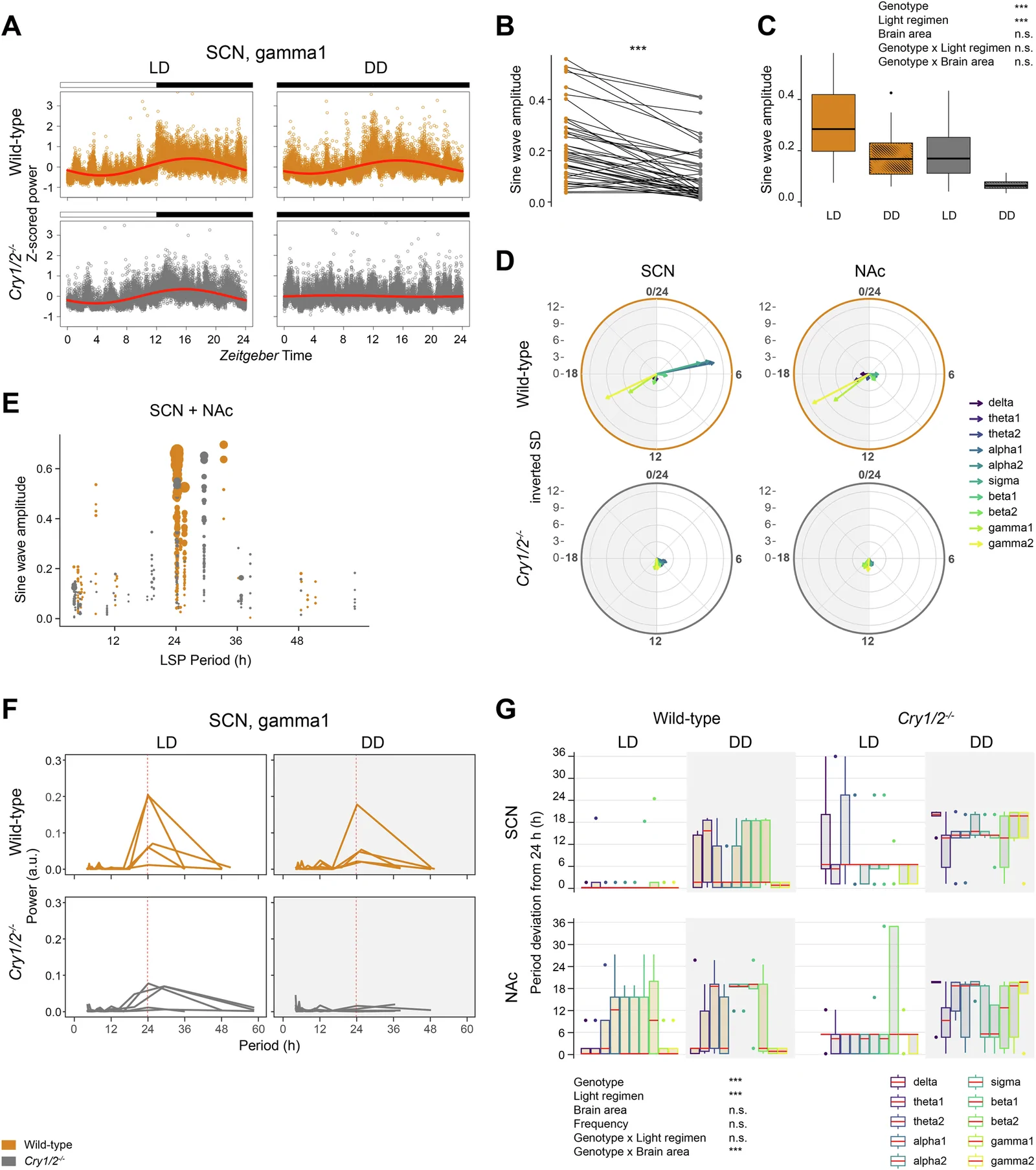
Endogenous and environmental circadian rhythms together generate and shape 24-hour rhythmicity of LFP activity and its low-frequency components. **A** Linear sine wave fit for pooled z-scored 24-h gamma1 SCN activity of all animals. **B** Comparison of wild-type and *Cry1/2*^*−/−*^ sine wave amplitudes of z-scored data. Individual connected points represent matched frequency – light regimen – brain area pairs. **C** As in (**B**), but boxplots for both genotypes in LD and DD. **D** 24-hour circular graphs with arrows pointing at the averaged *Zeitgeber* time of the highest amplitude of individual animals’ fitted sine waves. Arrow length indicates inverted standard deviation. **E** Timing of Lomb-Scargle peaks and matching sine wave amplitudes of individual animals for every frequency – light regimen – brain area pair. Point size corresponds to power of respective Lomb-Scargle peak. **F** Lomb-Scargle periodograms of gamma1 time series of all individual animals in the SCN. **G** Absolute deviation of largest Lomb-Scargle power peaks from 24 h. Median values are shown in red. Asterisks in (**B**) refer to student’s t-test, in (**C**) to Type II ANOVA, in (**G**) to a general multivariate regression model.

**Fig. 3:**
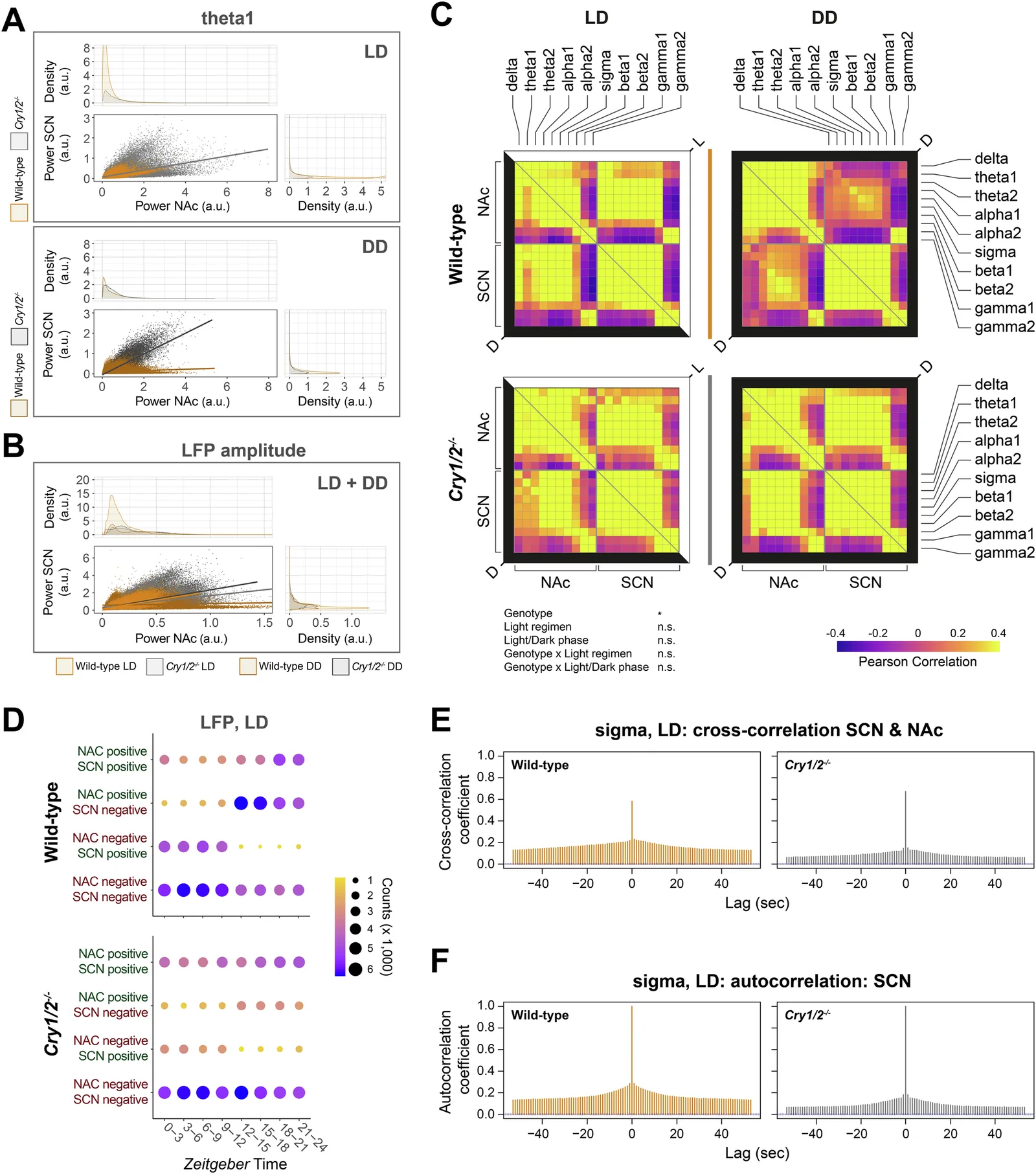
Endogenous circadian clocks determine the temporal sequence of LFP activities and its low-frequency components across brain regions. **A** Pearson correlation of pooled theta1 activity from all animals with density plots for point clouds. **B** Pearson correlation of pooled LFP amplitude from all animals. **C** Pearson correlation color matrix for frequency pairs from each genotype’s averaged data. Correlation coefficients were obtained for the first (ZT0-12, subjective day, upper right corner) and second half (ZT12-0, subjective night, lower left corner) of the 24-h day. **D** Correlation count plot for z-scored LFP amplitude (for graphical explanation, see Suppl. Fig. 4B) between both regions under LD. Occurrences of datapoints for respective conditions within each 3-hour time bin were counted, point size and color refer to the number of occurrences. **E** Cross-correlation of sigma activity between SCN and NAc under LD. Each bar refers to a correlation value with a multiple of a 1-second time-lag. Values on the x-axis signify the lag in seconds for the corresponding correlation value. **F** Autocorrelation of sigma activity in the SCN under LD. Depiction as in (**E**). Asterisks in (**C**) refer to a Type II ANOVA of unpooled coherence coefficients (correlation values only of matching frequency bands) of all individual animals.

**Fig. 4:**
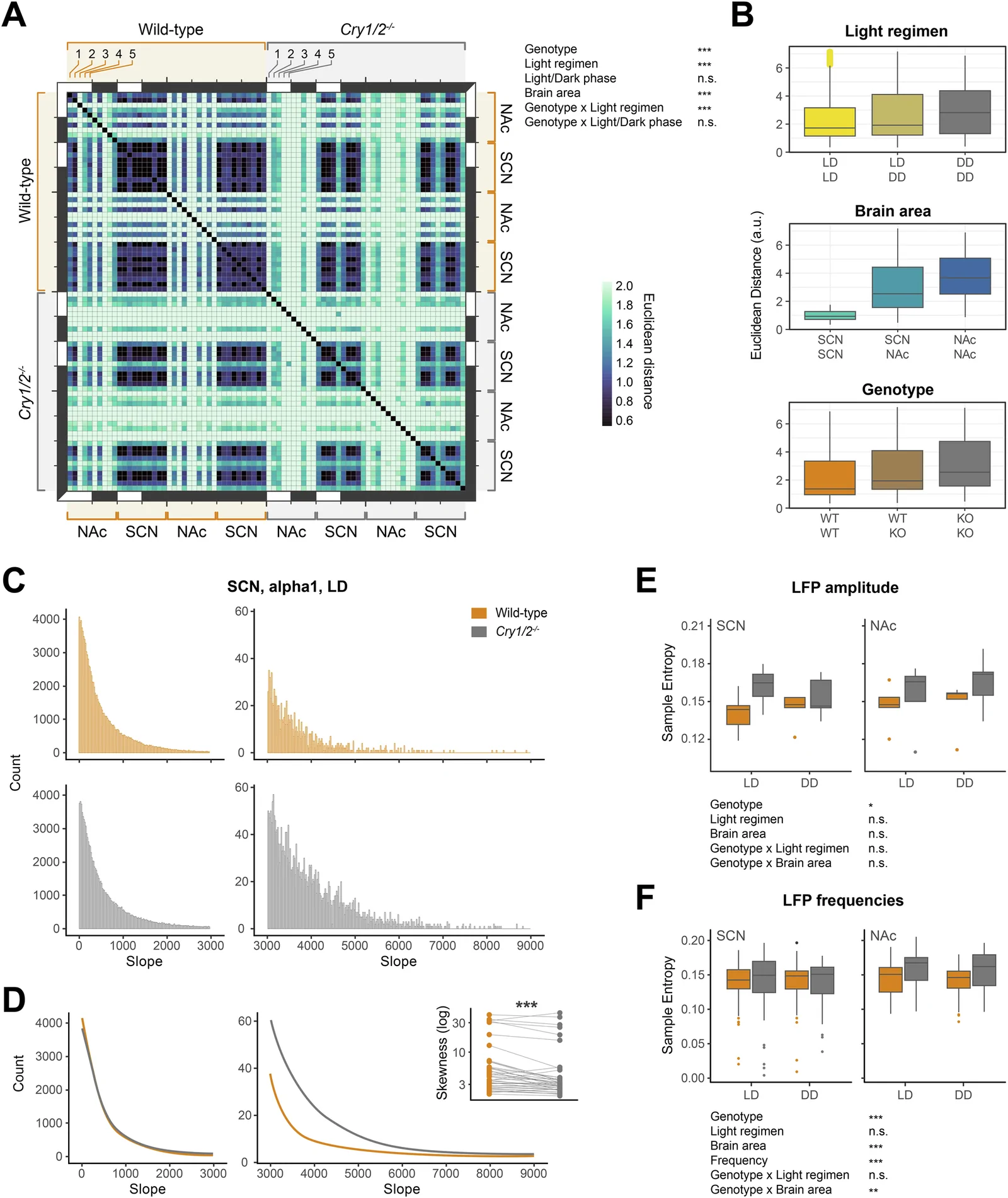
Endogenous and environmental circadian rhythms together increase uniformity and steadiness of low-frequency LFP component activities within and between brain regions and between animals. **A** Euclidean distance matrix for distances (a.u.) between every combination of every time series from single animals (for each genotype listed once as numbers 1 to 5), averaged across all frequencies. **B** Quantification of all Euclidean distances, grouped by the different variables. **C** Histograms of all slope parameters of SCN LD alpha1 time series, counting occurrences within a binwidth of 20. **D** Curve fit to the histograms in (**C**). Inlay panel: comparison of skewness values of slope parameter histograms for all frequency – light regimen – brain area pairs. **E** Sample entropy of all LFP amplitude time series. **F** Sample entropy of frequency time series pooled for all frequencies. Asterisks in (**B**) refer to a general multivariate regression model, in (**E**) and (**F**) to Type II ANOVA, in (**D**) to a paired Wilcoxon rank-sum test.

Data for 24 h spectral band spectrograms in Fig. 1A and S4A, 24 h relative LFP amplitude time series in Fig. S4B, sine wave analyses in Fig. 2A–D and S5A–D, correlation count plots in Fig. 3D and S6C, slope analysis in Fig. 4C, D and S8B, and mean frequency time series in Fig. S4E were z-scored for each individual frequency band or LFP time series for every animal individually (by subtraction of mean and division by standard deviation). This was done to compare phenomena beyond absolute values of either spectral power or LFP amplitude, but instead relative to activity levels throughout the 24-hour day.

For spectrogram block analysis that is displayed and quantified in Fig. 1B, C and S4C, after z-scoring (leading to the mean being 0) all values above 0 were equated to 1000, all values below 0 to −1000. Using R function *rollmeanr* of the *zoo* package [48], the rolling average was calculated over a window of 250 values. Afterwards, again all values above 0 were equated to 1000, all values below 0 to −1000. This was done in order to quantitatively enhance the contrast between relatively high and low power events and reproduce the impression of activity blocks visually observed from the spectrograms that we were then able to quantify. Colors correspond to spectrograms as depicted in Fig. 1A. R’s *base* function *rle* was then used to count the number of blocks per frequency band and animal.

#### Sine wave fit

For sine wave fit in Fig. 2A and S5A, B, a linear fit to the pooled data of each genotype within the same brain area and light regimen was carried out using:

$${{\rm{A}}} \, {\sim} \, {\sin} (2 \uppi / 24 * {Zeitgeber} \, {{\rm{time}}}) + {\cos} (2 \uppi / 24 * {Zeitgeber} \, {{\rm{time}}})$$

with A being either a frequency band’s spectral or a LFP 4-second binned (pooled) time series over 24 h. To achieve single animal resolution for our corresponding quantitative descriptions of amplitudes in Fig. 2B–E and S5C, D, we derived likewise calculated fits for each animal’s individual time series in respective brain areas and light regimens by then detecting each curve’s maximum and the corresponding *Zeitgeber* time.

#### Lomb-Scargle periodogram

Lomb-Scargle periodogram data displayed in Fig. 2E–G and S5E, which estimates frequency spectra of time series based on a least-squares fit of sinusoids, was created using *lsp* function from *lomb* package [49] and obtained from full and undisrupted recording periods (excluding habituation) with a duration of up to 72 h of the 4-second binned spectral and LFP data. Derived quantitative comparisons in Fig. 2E, 2G, and S5E utilized exclusively each animal’s highest peak of the respective Lomb-Scargle periodogram data, regardless of its amplitude.

#### Linear correlation

Linear correlations were carried out as Pearson correlation between a frequency band’s spectral or a LFP 4-second binned time series of both brain areas under a certain light regimen, pooled for each genotype as shown in Fig. 3A, B or for individual representative animals shown in Fig. S6A. Correlation matrices in Fig. 3C and S6B were obtained by calculating the Pearson correlation coefficient for different combinations of frequency bands within the same light regimen for each animal individually and then averaging over corresponding values. The creation of the correlation count plot in Fig. 3D and its four categories is explained in Fig. S6C.

#### Cross- and autocorrelation

Cross- and autocorrelations displayed in Fig. 3E, F and S6D–G were calculated using R function *ccf* of the *tseries* package [50]. Each correlation was calculated using 24-hour spectral or LFP data for LD and DD (and in case of autocorrelation each SCN and NAc) separately and averaged over all animals within the same genotype.

For quantitative comparison of correlations shown in Fig. S6G, we took all cross- and autocorrelation values, respectively, for every multiple of 1-second lag (as displayed in the plots described before) and averaged them within four time bins (two with negative, two with positive lag) for each animal individually from 1-second binned recordings of either spectral frequency bands or LFP signal. This was done for each light regimen and, in the case of autocorrelation, for each brain area individually.

#### Coherence measurement

Coherence measurements of Fig. S7 were performed using R function *coh* of the package *seewave* [51]. More specifically, raw LFP traces at four different *Zeitgeber* times (ZT0, 6, 12, and 18) over the course of 6 min each were divided into three bins of 108 s with an overlap of 36 s each. The coherence at each *Zeitgeber* time was then averaged over the three bins and plotted using linear smoothing (implemented in *geom_smooth* within *ggplot*) with 95% error bands, averaged for the different parameters specified in Fig. S7.

#### Euclidean distance

Euclidean distance for each time series pair was calculated using the formula

$$f\left(A,B\right)={\sum}_{{ZT}0}^{{ZT}24}\sqrt{{({B}_{{x}_{{ZTn}}}-{A}_{{x}_{{ZTn}}})}^{2}}+{({B}_{{y}_{{ZTn}}}-{A}_{{y}_{{ZTn}}})}^{2}$$

with A and B being two time series and, for example, ${A}_{{y}_{{ZTn}}}$ being the y-coordinate of a defined time point ZTn of time series A, such that only the distance of corresponding time points was calculated. Our final metric reduces this distance between each combination of compared time series to one single measure with higher values indicating higher distance and lower values lower distance. We show one matrix that averages all frequencies in Fig. 4A and an example matrix for gamma1 in Fig. S8A. For quantitative comparisons in Fig. 4B, the entire data was split into three groups for each binary variable (light regimen, brain area, or genotype, respectively). For example, for light regimen all Euclidean distances between pairs of two time series both under LD would form the first group, all Euclidean distances between pairs of two time series where one was under LD and the other under DD form the second group, and all Euclidean distances between pairs of two time series both under DD form the third group.

**Fig. 5:**
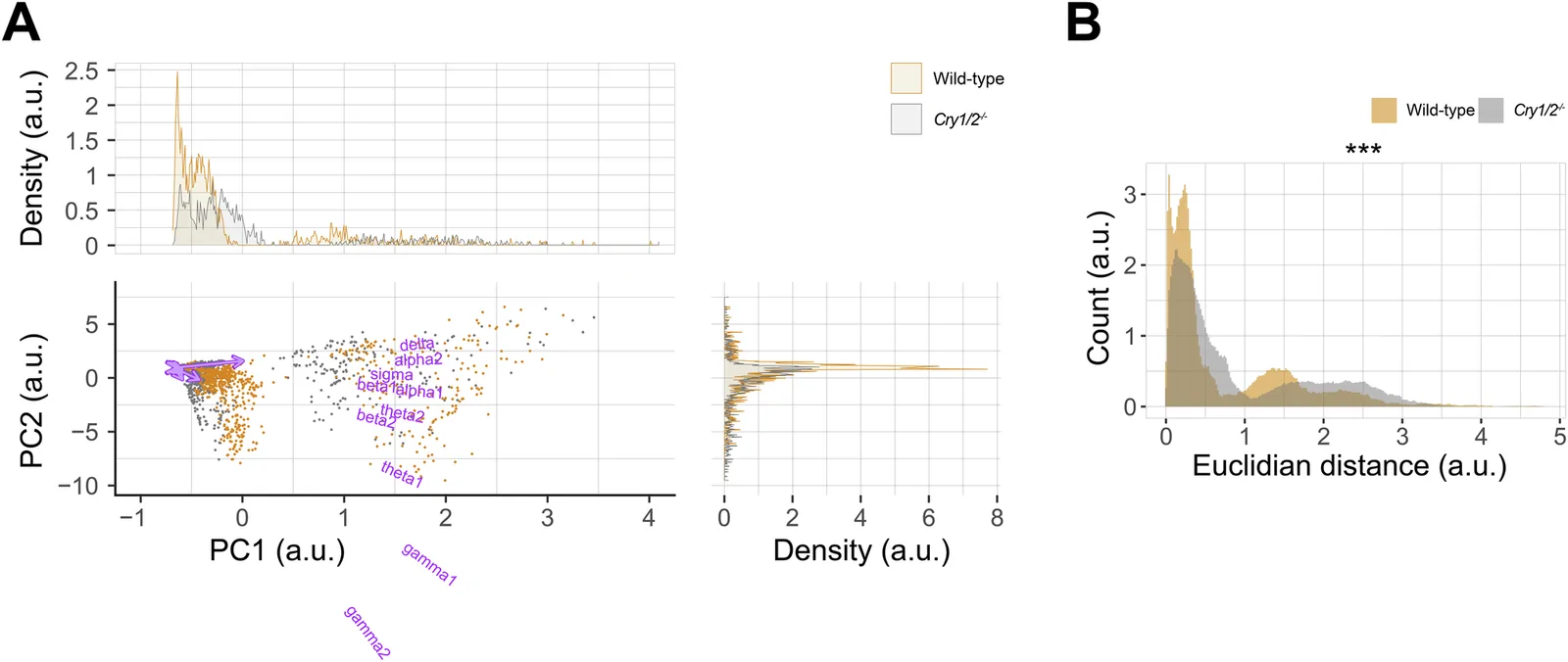
Disruption of endogenous circadian rhythms increases the variance of LFP constituents in multi-dimensional analysis. **A** PC1 vs PC2 of a PCA of the complete dataset of one-hour binned activity with variable (frequency) arrows and density plots of point clouds. **B** Histogram of distribution of Euclidean distance values for all combinations of datapoints within each genotype shown in (**A**). Asterisks in (**B**) refer to a two-sample Kolmogorov-Smirnov test.

For Euclidean distance of PCA results depicted in Fig. 5B, all possible pair-wise combinations of datapoints for principal components 1 and 2 (PC1 and PC2) within each genotype were used accordingly. The occurrence of obtained values was displayed as histograms with a binwidth of 2e-10.

#### Slope analysis

Slope values were calculated for every two consecutive z-scored time series points by dividing their difference in spectral power by the time bin step size. Occurrences of slope values were then counted and displayed as histogram in Fig. 4C and S8B. X-axes were truncated for values between 0 and 3,000 (left plot) and values between 3,000 and 9,000 (right plot). Skewness of histogram distributions was assessed using the *skewness* function of R package *moments* [52] for every frequency-light regimen-brain area combination and compared between genotypes in Fig. 4D.

#### Sample entropy

Sample entropy [53] was calculated using the *FastSampEn* function of the *TSEntropies* package [54] and, briefly, assesses complexity and self-similarity of time series by identifying similar epochs and assigning higher values indicating lower regularity and lower values indicating increased self-similarity. This analysis yields a single value for each individual 24-hour time series analyzed. These single measures are displayed as boxplots for all 4-second binned LFP in Fig. 4E and for the pooled frequency time series in Fig. 4F, separated for brain area, light regimen, and genotype.

#### Granger causality

Granger causality was calculated for the compiled time series of each genotype under each light regimen in each brain region using *grangertest* of the package *lmtest* [55] with results displayed in Table 1. The function carries out a Wald test comparing whether a time series A is only explained by (the lags of) A itself or by (the lags of) both time series A and B.

**Table 1:** Granger causality shows prediction accuracy more strongly in the direction from SCN to NAc.

	Wild-type				*Cry1/2*^−/−^			
	SCN → NAc		NAc → SCN		SCN → NAc		NAc → SCN	
	p-value	summary	p-value	summary	p-value	summary	p-value	summary
LD	< 2E-16	***	< 2E-16	***	< 2E-16	***	0.3386	n.s.
DD	0.0001637	***	0.4216	n.s.	< 2E-16	***	0.2793	n.s.

Granger causality for each genotype’s pooled time series between the two brain regions in both directions with each p-value of F-statistics representing the likelihood of finding causality if no causality was present between the samples.

#### PCA

For Principal Component Analysis, the *prcomp* function from R package *stats* was used. Briefly, PCA transforms data into a new coordinate system in a way that its greatest variation is captured along the axes. Time bins were treated as observations, spectral power bands as variables. Plotting was carried out using *ggplot* and *ggbiplot* from the *ggbiplot* package [56], the latter for overlay of the variable (frequency) arrows. Figure 5A and S9B show results for one-hour binned activity, while Fig. S9A shows results for 4-second binned data of all frequency bands. Euclidean distance was calculated using the 1-hour binned data for every possible datapoint pair within genotypes.

#### Fold-change heatmap

**Fig. 6:**
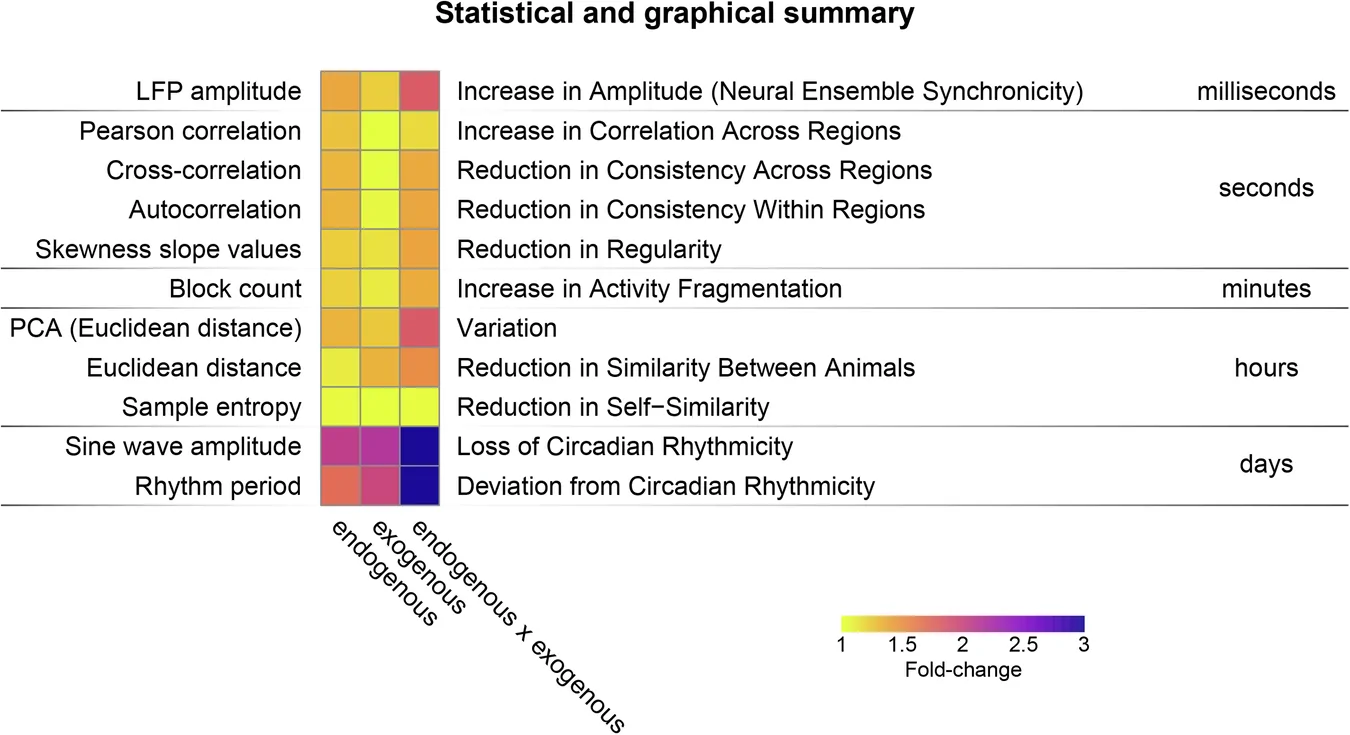
Disruption of endogenous and exogenous clocks affects properties of LFP and its spectral content across all time scales. Heatmap summarizing the parametrized main findings of our study as displayed in Figs. 1–5. Values were derived by pooling measures described on the left side of the heatmap for all frequency bands (except for LFP amplitude) and both brain regions (where applicable) within four groups: wild-type and *Cry1/2*^*−/−*^ animals under LD and DD each; and calculating the mean within these four groups. We then used wild-type mice under LD as reference to calculate relative fold-changes in *Cry1/2*^*−/−*^ animals under LD (endogenous), wild-types under DD (exogenous) and *Cry1/2*^*−/−*^ animals under DD (endogenous × exogenous). All measures displayed here are represented throughout the other figures. Descriptions on the right side of the heatmap are a suggestion for how to understand the obtained measures in a neurophysiologically meaningful way.

For every measure obtained in this study, we calculated fold-changes relative to wild-type animals under LD and displayed them as heatmap in Fig. 6. LFP amplitude derives its values from Fig. 1E, Pearson correlation from Fig. 3C, Cross-correlation and Autocorrelation from Fig. S6G, Skewness slope values from Fig. 4D, Block count from Fig. 1C, PCA (Euclidean Distance) from Fig. 5B, Euclidean distance from Fig. 4A, Sample entropy from Fig. 4F, Sine wave amplitude from Fig. 2C, and Period deviation from Fig. 2G. In each case, all available data from all frequencies and, where applicable, both brain regions were averaged for each of these four groups: wild-type under LD, wild-type under DD, *Cry1/2*^*−/−*^ under LD, and *Cry1/2*^*−/−*^ under DD. Relative changes to wild-type under LD were then calculated for the other three groups. Where the relative change was negative, directionality of the change was inverted to paint a coherent picture in the heatmap.

## Results

### Disruptions of endogenous and environmental circadian rhythms increase fragmentation of LFP activity over 24h

We recorded LFP signals with stereotactically placed electrical probes in the SCN and NAc of five wild-type and five *Cry1/2*^*−/−*^ animals (Fig. S1A, B). Raw traces of our LFP recordings and the corresponding spectrogram show contamination-free recordings under both LD and DD (Fig. S2A, B) that are stable in amplitude and quality over the entire recording period (Fig. S2C). To ensure that we are describing real underlying neuronal oscillatory phenomena and not only irregular transients with high amplitude [57], we computed the continuous wavelet transform [44, 45] for selected sections of our raw LFP traces using Morlet wavelets (Fig. S3A). The captured LFP signals contain neural oscillatory events occurring in the entire frequency range analyzed throughout our study (and less so below and above this range). However, we identified two frequencies which stood out in displaying pronounced peaks in the time-frequency plane: one in the alpha1 and one in the gamma2 range (Fig. S3A). Oscillations at these two frequencies largely extend over ± 10 Hz, with only minor differences between the two genotypes (Fig. S3B). In exemplary traces of neural oscillatory events, we observe that in the alpha1 range they have a longer duration, while those in the gamma2 range are rather short (Fig. 1A, S3C) which is confirmed in a density plot of their event duration distribution (Fig. S3D). Properties of neural oscillatory events show little differences between the two brain areas in the alpha and remarkable differences in the gamma range, with the SCN displaying shorter durations (Fig. S3D) and more low-power events (Fig. S3E). Intervals between events of both frequencies, however, depend on the light regimen (shorter during LD) (Fig. S3D). Finally, wild-type animals display more high-power events in the gamma range, while *Cry1/2*^*−/−*^ mice show more of them in the alpha range (Fig. S3E). These initial results demonstrate that our recordings capture neural oscillatory events instead of just spectral noise. Events occur especially pronounced in the alpha and gamma range. Our results also show how these events are modulated differentially contingent on the brain area from which they are recorded and on diurnal rhythms, with differences in neural oscillatory power being dependent on endogenous clock function and intervals being modulated by the light regimen.

Exemplary raw traces of detected neural oscillatory events in the SCN for animal C1 (Fig. 1A) emphasize that our spectral data is capturing underlying neuronal ensemble phenomena that constitute the basis for our further analyses. To now obtain a general impression of 24-hour dynamics of the LFP time series of the SCN and the NAc under the influence of endogenous and exogenous rhythms, we used a graphical visualization of the z-scored activity within all frequency bands and fast Fourier transformed LFP amplitude of an exemplary ZT0-ZT24 epoch for each animal under LD and DD conditions. For both brain regions, these graphs show a rough division of frequency specific activity and LFP amplitude into homogenous blocks (Fig. 1A, S4A, B). Under LD conditions, a clear demarcation line of frequency distribution is visible at ZT12 for all wild-type animals, defined by a switch from relatively low to overall higher gamma activity (and vice versa in the remaining frequency bands) (Fig. 1A top left, S4A top left). In the same animals, the day-night switch between frequency specific activity is preserved under DD conditions, however due to individual free-running periods of the animals without a comparably clear line after the first 12 h of the recording section shown (Fig. 1A top right, S4A top right). Also, in *Cry1/2*^*−/−*^ mice, the frequency-specific activity under LD conditions appears to be different in the light and dark phases of the LD cycle, although no such clearly defined demarcation line as in wild-type animals is evident (Fig. 1A bottom left, S4A bottom left). However, in DD *Cry1/2*^*−/−*^ animals do not display any obvious diurnal patterns (Fig. 1A bottom right, S4A bottom right). Beyond that, the recorded signals very accurately reflect changes between increases and decreases of locomotor activity, as shown for animals W5 and C5 (Fig. 1A, S4A).

The optical impression of a demarcation line between light and dark or active and inactive phase arises primarily from different relative activity distribution patterns within frequency bands specific to the respective time of day, which appear especially in wild-type animals in the form of temporally extended, homogeneous blocks and are contrasted by a fragmentation of blocks in *Cry1/2*^*−/−*^ animals. To be able to quantify this merely visual phenomenon, after binary splitting of z-scored values, we calculated the rolling average of each animal’s time series and, again, split the obtained values with regards to their relation to the mean. This reproduced the visually identified distribution pattern of blocks (Fig. 1B) where each block type corresponds to relatively high or low spectral power or LFP amplitude, respectively. Counting the number of blocks confirms that wild-type animals have fewer blocks than *Cry1/2*^*−/−*^ mice in LD, indicating a less fragmented distribution of frequency activity (Fig. 1C) and LFP amplitude (Fig. S4C). In contrast, *Cry1/2*^*−/−*^ animals more often alternate between the two block types and both types occur with the same probability regardless of the time of day. Furthermore, the analysis shows that, independently of genotype, the light regimen significantly affects the number of blocks with fewer blocks in LD than in DD, and that during the light phase (LD) or subjective day (DD) the frequency activity is more fragmented than during the dark phase / subjective night.

### Disruption of endogenous circadian rhythms increases LFP amplitude

LFPs reflect local electrical currents caused by aggregation of neuronal ensemble activity [23]. Hence, their intensity is hypothesized to be indicative of the level of synchronicity of these ensembles, with higher intensities indicating higher synchronicity [58, 59].

Depiction of LFP amplitude of both brain areas also shows no diurnal pattern in *Cry1/2*^*−/−*^ animals, but in wild-type animals, especially under LD conditions. Interestingly, their LFP amplitude in SCN and NAc shows an antiphasic circadian rhythm (Fig. S4B). Accordingly, averaged one-hour bins of the fast Fourier transformed LFP signal show a dip around ZT12 in the SCN that is weakly preserved under DD (Fig. 1D, S4D), but an increase around ZT12 in the NAc. The similar applies to the investigation of frequency-specific activity, e.g., gamma1 (Fig. S4E). Here, wild-type animals, especially in contrast to *Cry1/2*^*−/−*^ animals under DD, consistently show narrow standard deviation margins and a steep increase or decrease in activity around ZT12.

Moreover, quantifying the area under the curve of the fast Fourier transformed LFP signal shows higher values for *Cry1/2*^*−/−*^ animals under both light regimens in the SCN as well as the NAc (Fig. 1E), implying higher synchronicity of local neuronal activity when the endogenous clock is disrupted. Furthermore, the analysis shows that LFP amplitude in the NAc is consistently higher than that of the SCN regardless of genotype and light regimen.

### Endogenous and environmental circadian rhythms together generate and shape 24-hour rhythmicity of LFP activity and its low-frequency components

In order to investigate more precisely and systematically the influence of endogenous and environmental diurnal rhythms on electrical properties of neurons and their circadian rhythmicity, we carried out linear fitting of a 24-hour sine wave for the different z-scored time series to compare the amplitudes as a surrogate for the strength of 24-h rhythmicity (Fig. 2A, S5A, B; for statistics, see Suppl. File). Comparison of all amplitudes of these wave fits between wild-type and *Cry1/2*^*−/−*^ mice for all single frequencies (for SCN and NAc under LD and DD each) shows that, with a few exceptions, the amplitudes of *Cry1/2*^*−/−*^ mice are always lower than those of wild-type mice, with some of their amplitudes being close to 0 (Fig. 2B and S5C). The highest amplitudes of 24-h rhythms are found in wild-type animals in LD (Fig. 2C). In DD, when only the influence of endogenous rhythms remains, the amplitudes of the individual frequencies of wild-type mice decrease significantly. Interestingly, the magnitude of these amplitudes is then very similar to that of *Cry1/2*^*−/−*^ mice in LD, where only exogenous rhythmic signals are present – suggesting that endogenous and exogenous time signals each have a similarly strong influence on circadian neuronal activity rhythms. When both rhythmic influences, endogenous and exogenous, are omitted, as is the case in *Cry1/2*^*−/−*^ mice in DD, the amplitudes of the frequencies hardly exceed 0 overall.

Interestingly, circular plotting of the 24-h sine wave fit phase shows that in the SCN and the NAc of wild-type mice some frequencies predominantly peak between ZT15-16, i.e., in the activity phase (Fig. 2D). This effect is predominantly carried by gamma frequency bands, which are associated with attention and consciousness in the cortex, offering a potential explanation to why their peak occurs during the animals’ active phase [60, 61]. Besides, there is another cluster in the SCN in the opposite phase at around ZT5, in which mainly theta, alpha, and sigma frequencies are present, which are associated with decreased activity, relaxation, and sleep [62]. In contrast, in *Cry1/2*^*−/−*^ mice, neither in the SCN nor in the NAc are times discernible at which certain frequency peaks are particularly pronounced. With additional consideration of the light regimen, these patterns are found to be essentially maintained in wild-type mice, but with less pronounced peak clustering in DD (Fig. S5D). Similar to wild-type mice but with less pronounced patterning, the peaks of gamma frequencies in the SCN of *Cry1/2*^*−/−*^ mice under LD are clustered between ZT15-16 and of sigma at around ZT6. This pattern is absent in SCN under DD and in NAc under both LD and DD.

As the sine wave fit inherently assumes 24-h rhythmicity, we additionally drew Lomb-Scargle periodograms for each animal for every frequency under both light regimens [63] to investigate which other periods naturally occur. The combined examination of 24-h sine wave fit amplitudes and the timing and power of the detected dominant periods of the Lomb-Scargle analysis illustrates that the periods of wild-type animals predominantly cluster in the circadian range, i.e., around 24 h, whereas *Cry1/2*^*−/−*^ animals show a broader period range and less powerful rhythms (Fig. 2E). With explicit consideration of exogenous influences by the light regimen, it shows that rhythms with circadian period and strong power can especially be found in wild-type animals in LD and with lower power in DD, whereas they are hardly present in *Cry1/2*^*−/−*^ animals, especially in DD, here exemplified for gamma1 power of the SCN (Fig. 2F). Accordingly, the dominant period of all SCN frequencies of wild-type mice in LD almost never deviates from 24 h, and predominantly deviates only little in DD towards periods slightly longer than 24 h (Fig. 2G, top left). In contrast, dominant periods in *Cry1/2*^*−/−*^ SCNs mostly differ by about 5 h in LD and deviate so much that they no longer fall within the range of circadian periods in DD (Fig. 2G, top right), although the animals show strong 24-h masking in behavior. The NAc exhibits similar patterns (Fig. 2G, bottom), however, unlike in the SCN, stronger deviations from 24 h are present in wild-type animals in both LD and DD, which is especially evident in alpha2, sigma, and beta1 frequencies in DD, whose periods deviate nearly 20 h from 24-h rhythms. Similarly, LFP amplitude of both brain regions deviates strongly from 24-h rhythmicity in *Cry1/2*^*−/−*^ animals, especially in DD, but less so in wild-type animals (Fig. S5E). Thus, again, both genotype and light regimen have a striking effect on electrical activity rhythms within brain areas, with loss of endogenous and exogenous timing cues each leading to greater variance in rhythm periods.

### Endogenous circadian clocks determine the temporal sequence of LFP activities and its low-frequency components across brain regions

As we recorded local field potentials from the SCN and the NAc of each mouse simultaneously, we were able to assess correlation of activity and carrier waves between the two brain regions. Using different scaling approaches (linear, exponential, and logarithmic) to our variables led to comparable R-squared values in our correlational analysis. Because of the cloud-like distribution of data points, and its biological meaningfulness and ease of understanding, we chose a linear model.

Under LD, we observe similar positive correlations between SCN and NAc in both wild-type and *Cry1/2*^*−/−*^ animals, but with higher density of point clusters in wild-type animals (Fig. 3A; for statistics, see Suppl. File). However, under DD conditions, the linear regression lines clearly differ between wild-type and *Cry1/2*^*−/−*^ animals with a very pronounced positive correlation between SCN and NAc in *Cry1/2*^*−/−*^ animals and a flat correlation line in wild-type animals. These findings, here exemplified by theta1, are consistent across the various frequency bands, and for LFP amplitude in averaged and individual representative animals (Fig. 3B, S6A, S6G; for statistics, see Suppl. File). To get an overview over all frequency pair correlation values between and within SCN and NAc, we then created a correlation matrix for each genotype, light regimen, and (subjective) daytime, with the upper right side displaying (subjective) daytime and the lower left side (subjective) nighttime values. While the notion of only smaller discrepancies between wild-type and *Cry1/2*^*−/−*^ animals in LD is confirmed across frequency pairs, in DD smaller linear correlation values between SCN and NAc are prominent in wild-type animals, but not in *Cry1/2*^*−/−*^ animals (Fig. 3C, S6B). Statistical analysis of all coherence coefficients (within our discrete bands) further confirms a significant difference of correlation values between the two genotypes.

Next, we asked whether the correlation patterns change throughout the day, i.e., as a function of *Zeitgeber* time. For this, we correlated the z-scored LFP amplitude of SCN and NAc and counted the occurrence of four possible correlation constellations of high and low SCN and NAc LFP amplitude (Fig. S6C for visualization) according to their appearance within eight 3-hour *Zeitgeber* time windows covering 24 h. Interestingly, wild-type animals consistently display a distinct time course of correlation values with relatively higher SCN activity accompanied by lower NAc activity during daytime and lower SCN activity accompanied by higher NAc activity during nighttime (Fig. 3D). In contrast, this circadian pattern is almost completely lost in *Cry1/2*^*−/−*^ animals in which higher or lower SCN and NAc activity predominantly occur together.

Communication between brain areas also occurs lagged. Besides correlation of neuronal activity at the same timepoint, certain state transitions might only be covered by larger time intervals [64]. Therefore, we sought to investigate cross-correlations within 1-second bins between SCN and NAc activity covering a time lag of almost 60 s, here exemplified by sigma in LD (Fig. 3E) and LFP amplitude in DD (Fig. S6D). In wild-type animals, highest correlation between SCN and NAc occurs at the same time point, i.e., time lag 0. However, at time points before and after, a relatively high correlation between SCN and NAc is also evident. In contrast, *Cry1/2*^*−/−*^ animals show a sharp and compared to wild-type animals higher peak at 0 s time lag and, additionally, all time points before and after appear flatter. Hence, within the same time bin, electrical activity of the SCN and the NAc is excessively synchronized in *Cry1/2*^*−/−*^ animals, whereas in wild-type animals it is more non-synchronous meaning that correlative levels of neuronal activity between the two brain regions remain stable for a longer time period. The same pattern is also recognizable in autocorrelations within the same brain region, here shown for sigma in LD (Fig. 3F, S6D, E) and LFP amplitude in LD in the NAc (Fig. S6D) and is stable across frequencies with the only exception being delta in LD (not displayed). The described patterns and strengths of correlation are not observable in a negative control correlation (Fig. S6F). Finally, this pattern is stable across frequencies in both light regimens and can be quantified for both cross- and autocorrelation (Fig. S6G).

To describe the synchrony of carrier waves between SCN and NAc more detailed and beyond spectral power of frequency bands, we also assessed coherence measurements [65] of our raw LFP traces which assess linear association as a degree of synchronous oscillations between two signals such as time series. Averaged traces over LD and DD as well as the four *Zeitgeber* times assessed (ZT0, 6, 12, and 18) for all individual animals reveal clear differences between the two genotypes. Most noticeably, *Cry1/2*^*−/−*^ animals show a unified peak around 10 Hz (Fig. S7 top) which is even more pronounced under DD. These observations confirm our previously shown results of excessive synchronization of activity between SCN and NAc in absence of endogenous and exogenous clocks from linear correlations of the binned spectral power (Fig. 3A, C). Pooling measurements across genotypes and light regimens for individual animals further validates the 10 Hz peak in *Cry1/2*^*−/−*^ animals (Fig. S7 middle). Adding *Zeitgeber* time to this depiction shows that of all variables analyzed, genotype is the best predictor of the coherence measurement curve’s shape (Fig. S7 bottom).

Taken together, correlation analyses display remarkable differences between wild-type and *Cry1/2*^*−/−*^ animals. Especially, but not exclusively under DD conditions, *Cry1/2*^*−/−*^ animals show higher correlation values between SCN and NAc that we interpret as an excessive synchronization of activity in the absence of a circadian pacemaker. Between- and within-region electrical activity of wild-type animals, in contrast, appears more non-synchronous and coordinated over the course of many seconds. Thus, our data exposes the presence of an endogenous circadian clock as a powerful determinant on consistency of activity between and within two regions of the brain.

### Endogenous and environmental circadian rhythms together increase uniformity and steadiness of low-frequency LFP component activities within and between brain regions and between animals

To further interrogate the influence of circadian rhythms on the nature of 24-h time course activity of LFP components, we obtained an estimate of the similarity of frequency band time series 1) across both genotypes, 2) across the different light regimens, 3) across SCN and NAc, and 4) across individual animals. Cross- and autocorrelation, as shown above, have revealed that neuronal activity over the course of many seconds in wild-type animals shows more consistency of concerted activity between and within brain areas when compared to *Cry1/2*^*−/−*^ animals. In order to obtain a measure for regularity and similarity of the time series over the course of an entire 24-h period, as a next step, we therefore calculated the Euclidean distance between individual time series. Euclidean distance provides information about how close or far data points of two individual time series are from each other at every given time point, i.e., how similar the two curves are. We assessed the Euclidean distance between all the different combinations of time series of all individual animals for each brain area, light regimen, and light/dark phase as one single measure per time series pair each. A matrix was generated for every frequency band individually (as shown for gamma1 in Fig. S8A). We then calculated one average value from all frequency matrices for every datapoint to get a comprehensive matrix (Fig. 4A). Furthermore, we extracted the cumulative effects of our main variables (light regimen, brain region, and genotype) from the matrix (Fig. 4B). We see that regardless of genotype, the similarity of the time series depends significantly on the light regimen (Fig. 4B, top) and also on the brain area (Fig. 4B, middle). In LD, the time series are much more similar to each other than in DD and also more similar than in the LD-DD comparison. Likewise, Euclidean distances are very small for comparisons within the SCN, whereas they are much larger in the NAc, and the values of the SCN-NAc comparison are intermediate.

However, the most striking difference of the distance matrix constitutes an uneven color gradient from the upper left, where the values of the wild-type animals are clustered, to the lower right corner, where the *Cry1/2*^*−/−*^ data is located. This gradient is indicative of overall lower distance values, i.e., higher similarity, for within-wild-type comparisons than within-*Cry1/2*^*−/−*^ comparisons. A quantification of the respective distances comparing genotypes indeed confirms lower distances for wild-type than for *Cry1/2*^*−/−*^ animals (Fig. 4B, bottom). Interestingly, even when comparing wild-type with *Cry1/2*^*−/−*^ animals, the distances are lower than distances within the *Cry1/2*^*−/−*^ group. In addition, there is a relevant interaction of genotype and light regimen, with DD further increasing the distances in *Cry1/2*^*−/−*^ mice. Thus, taken together it appears that wild-type time series are generally steadier, even among each other, and less variable than those of *Cry1/2*^*−/−*^ animals.

A further surrogate for variability and regularity of a time series is the slope value for each consecutive time point pair, i.e., the (vertical) distance from one measuring point to the next. As a representative example, we have plotted the obtained slope values for all alpha1 time series in LD in the SCN as histograms, with slopes from 0–3,000 to visually show counts of occurrences of rather flat slopes, and from 3,000–9,000 to show the range of incidences of steeper slopes (Fig. 4C, S8B). We see that wild-type mice show quantitatively more flat slope values and fewer frequently steep slopes compared to *Cry1/2*^*−/−*^ animals, in which the overall distribution of slopes appears flatter and broader (Fig. 4D). This impression can be quantified and confirmed by the skewness of the histogram. Comparing the skewness values of the histograms for all frequency bands reveals that, overall, wild-type mice display higher skewness values than *Cry1/2*^*−/−*^ mice indicating that the transitions from one measuring point to the next within their time series are smoother than in *Cry1/2*^*−/−*^ animals (Fig. 4D, inserted panel).

Lastly, an additional parameter assessing self-similarity of time series is sample entropy, an analysis tool developed for the examination of biological time series [53]. This method identifies similar epochs and assigns a value to every time series with higher values indicating lower regularity and reduced self-similarity. Sample entropy analysis of both genotypes’ LFP time series of SCN and NAc shows higher values for *Cry1/2*^*−/−*^ animals than for wild-type animals equally for both brain regions and for LD and DD, again demonstrating endogenously lower regularity and less self-similarity in neuronal activity of *Cry1/2*^*−/−*^ animals (Fig. 4E). Comparing every frequency band’s time series of the two brain regions under both light regimens yields similar results, with significant differences between the two genotypes, but in this case also between brain areas and frequencies (Fig. 4F and S8C).

Beyond mere similarity of the individual time series, we also wanted to interrogate, whether the signals we had obtained from SCN and NAc contained any information that would relate them to one another beyond all previous analyses. We therefore determined Granger causality, a measure of the capability of one time series to predict another one, between the two brain regions’ averaged signals of each genotype. Our analysis reveals high explanatory power of the SCN LFP signal for the NAc LFP signal for both genotypes in both light regimens (Table 1, Fig. S8D). Interestingly, in contrast, the LFP signal of the NAc has only limited significant explanatory power for the SCN LFP signal. Only in wild-type animals in LD can the NAc LFP forecast LFP signal of the SCN, which is not the case in DD and in *Cry1/2*^*−/−*^ animals in both LD and DD. This result is interesting for several reasons, showing first that the regularity of neuronal activity in the SCN and NAc of wild-type mice is so high that the signal of one brain region can predict that of the other brain region. Second, it shows that this prediction is particularly accurate in the direction from SCN to NAc (which is true for both genotypes), indicating that the well-established hierarchical order between SCN and other brain regions seems to hold also at the level of non-circadian high frequency electrical activity. And third, rhythmic factors from the environment, here the light regimen, also have significant influence on the time-of-day dependent interplay between brain regions.

In conclusion, both endogenous and environmental circadian rhythms increase steadiness and smoothness of 24-h LFP time series and its frequency components within brain regions. Interestingly, this influence extends to the degree that the trajectories of LFP frequency components of individual wild-type animals and animals kept in LD are much more comparable to each other than to those of *Cry1/2*^*−/−*^ animals and those kept in DD.

### Disruption of endogenous circadian rhythms increases the variance of LFP constituents in multi-dimensional analysis

Finally, to account for the complex and multidimensional nature of the recordings, we evaluated the frequency components of each animal’s LFP signal for all time points at once using principal component analysis (PCA) [66]. PCA identifies an axis system in the multidimensional data space in which the directions of the axes, the principal components, correspond to the main directions in which the data vary, i.e., along which the data points separate best. Therefore, it allowed us to test, by which factors the variance within all our animals’ recorded datasets would actually be driven, and to evaluate the contribution of every frequency component. Displaying the separation of data along the first two principal components (with PC1 explaining 90.5% and PC2 5.7% of total variance in the data) shows a more pronounced data point separation for *Cry1/2*^*−/−*^ animals than for wild-type animals (Fig. 5A, S9A). Whereas data points of wild-type animals cluster strongly, data points of *Cry1/2*^*−/−*^ animals are more separated along the two axes, again confirming higher similarity of frequency band activity within individual wild-type animals and higher variance within *Cry1/2*^*−/−*^ animals. In order to actually quantify the difference in separation of datapoints between both genotypes, we measured the difference in variance by analysis of the Euclidean distance of every individual datapoint pair within both genotype groups (Fig. 5B). This analysis shows a significant discrepancy of the histogram distributions with more occurrences of high distances for *Cry1/2*^*−/−*^ animals. The separation of datapoints appears to be mainly driven by activity in the NAc, as it is NAc datapoints that separate more prominently along PC1 (Fig. S9B), and it is not exclusively caused by any single frequency as all frequency components contribute to the overall variance in similar fashion, indicated by the corresponding directions of the frequency band arrows (Fig. 5A, S9B). Only gamma1 and 2 appear to deviate from this general alignment, emphasizing that they might convey complementary information. Taken together, PCA shows that variance in our data is primarily driven by genotype whereas light regimen and brain area contribute to a lesser extent.

This increased variance of the spectral content of LFPs confirms the reduced self-similarity and consistency of neuronal ensemble activity in *Cry1/2*^*−/−*^ animals in the multidimensional variable space. While variance is higher amongst frequency bands in the NAc, those in the SCN in turn appear to be more consistent and homogenous in activity, especially in wild-type animals. PCA also shows that variation within the spectral content of LFPs largely arises along one dimension (PC1, evidenced by its high explanatory power of 90.5% and the alignment of most frequency arrows) – the dimension which also separates both genotypes best. As PCA incorporates all frequency components of any given point in time into one measure, it emphasizes the robustness of our reported findings.

### Disruption of endogenous and exogenous clocks affects properties of LFP and its spectral content across all time scales

Our deep analysis of local field potentials and their spectral content shows that disruption of endogenous and exogenous rhythms not only influences circadian but also ultradian processes across time scales. To summarize our findings, we calculated the mean of the most informative measures obtained in this study for our four experimental groups (wild-type and *Cry1/2*^*−/−*^ animals under LD and DD each) and displayed fold-changes in relation to wild-type mice under LD (Fig. 6). While cross- and autocorrelation are more dominantly affected by perturbation of endogenous clocks, exogenous clocks play a bigger role for Euclidean distance. The interaction of both clocks most prominently has its biggest influence on LFP amplitude, as well as sine wave amplitude and period deviation. This synopsis of our findings captures how our interventions differentially affect (the spectral content of) LFPs on different time scales.

## Discussion

We had based our study on three hypotheses. First, endogenous and exogenous 24-h rhythms and their interplay control circadian rhythms of neuronal activity within brain regions. Second, these factors moreover regulate the interaction between brain regions throughout the day. And third, the presence of endogenous and exogenous circadian rhythms influences the properties of neuronal activity beyond its circadian aspect.

Our data show a distinct diurnal distribution of homogenous blocks of neuronal activity and a well-defined 24-h rhythm of LFP amplitude in the SCN and the NAc. Interestingly, the blocks of neuronal activity correspond to phases of locomotor activity and inactivity and thus reflect the rhythmic and arrhythmic behavior of wild-type and *Cry1/2*^*−/−*^ mice, respectively. In wild-type animals, rhythms occur under LD and DD conditions, however, under DD, their amplitude is significantly reduced and the phase of individual LFP frequencies is more variable. In contrast, in *Cry1/2*^*−/−*^ animals, no rhythms can be detected in DD. However, LD-cycles are also able to induce rhythms in endogenously arrhythmic animals that are as pronounced in amplitude as those of wild-type mice in DD. This means that endogenous and exogenous timing cues are each similarly strong drivers of rhythms of neuronal activity that act additively in wild-type animals in LD.

Endogenous and environmental rhythms also influence the interplay between SCN and NAc. In LD, LFP intensities and individual LFP components of SCN and NAc correlate strongly in both genotypes. However, this correlation decreases remarkably in wild-type animals under DD but remains almost unchanged in *Cry1/2*^*−/−*^ animals. When these correlation values are assigned to binned *Zeitgeber* intervals over 24 h, it becomes clear that in wild-type mice, the NAc is often inactive during the light phase when the SCN is active and vice versa during the dark phase. This observation is consistent with the predominantly inhibitory role of the SCN [67, 68] and its increased activity in the light phase when nocturnal animals such as mice are inactive. Interestingly, in *Cry1/2*^*−/−*^ animals, despite rhythmic behavior in LD, this pattern of opposing activity of SCN and NAc is not evident and both brain regions are predominantly active or inactive simultaneously. Thus, while endogenous and exogenous factors can equally generate rhythmicity, endogenous clocks appear to be necessary for the precise choreography of SCN activity and activities of subordinate brain regions over the course of the day.

Most surprisingly, the presence of endogenous and exogenous circadian rhythms not only affects circadian baseline changes in neuronal activity within and between brain regions, but also their intrinsic nature and quality on a much shorter time scale. One of these changes is the constant increase of the fast Fourier transform of the LFP signal in *Cry1/2*^*−/−*^ animals which could be attributed to a higher synchronicity of local neuronal ensembles [58]. This assumption is supported, in one way, by the increased synchronization between SCN and NAc activity within the same time bin in *Cry1/2*^*−/−*^ animals. In contrast, LFP amplitude and LFP components of *Cry1/2*^*−/−*^ mice show decreased cross-correlation between SCN and NAc and autocorrelation within the two brain regions indicating a reduction in coherent state dependent activity over larger time intervals. Hence, modulated by endogenous rhythms, activity consistency between brain regions displays a differential regulation across different time scales. Overall, these results suggest that in the absence of endogenous circadian clocks, neuronal responses to each other are predominantly synchronized and to some extent more stereotypical in *Cry1/2*^*−/−*^ animals than in wild-type animals, where they are rather non-synchronous and generally more stable, as would be expected for physiological signals traveling through the healthy brain [69].

At the same time, the dynamics of the LFP signal and its frequency components over time appear to be less organized and well-structured in *Cry1/2*^*−/−*^ animals than in wild-type animals. Whereas in wild-type mice successive signals are rather similar, resulting in smoother time series, the differences from one measuring point to the next in the time series in *Cry1/2*^*−/−*^ mice are more drastic and the signal is characterized by greater entropy. Due to the increased steadiness of the time series in wild-type animals, they are more similar among each other and to *Cry1/2*^*−/−*^ animals than *Cry1/2*^*−/−*^ animals amongst themselves. Likewise, LD is able to reduce differences between time series. Thus, in the context of an animal population, circadian clocks together with environmental rhythms create more uniformity among individuals at the level of brain activity, which presumably translates into greater consistency of behavior across the day in the group. The robustness of this notion is further supported by the stronger separation of *Cry1/2*^*−/−*^ animals’ activity in the multidimensional dataspace and similar contributions of all frequency components to this variance. In general, we were able to observe consistent effects of disruptions of both endogenous and exogenous clocks on the different frequency components of our LFP recordings across all analyzed parameters.

To ensure that our recordings actually capture underlying neuronal ensemble phenomena, we screened them for neural oscillatory events. Being able to detect them throughout our data demonstrates that the captured spectral time series contain true LFP frequency components and therefore allowed us to study their modulation in the SCN and NAc over the course of 24 h in freely moving animals. In contrast, most previous research on neuronal ensemble activity and network oscillations has focused on cortex and hippocampus, only examined single frequency bands, explored only short time scales, and investigated their relation to specific tasks [27, 70–72].

Our results show that similar to the 24-hour regulation of spectral activity, the properties of the underlying neural oscillatory events in the SCN and the NAc are also modulated by circadian factors. Endogenous clocks have influence on the oscillatory power and the light regimen affects event intervals.

While our study design allowed us to identify remarkable differences across all frequency bands analyzed, alpha and gamma bands stand out in several spectral time series analyses and appear as pronounced frequencies in neural oscillatory events. This is of particular interest as they have already been extensively addressed in previous studies and our data can complement these results which have been rather inconsistent so far. One experimental paradigm revealed that alpha-gamma cross-frequency phase synchrony correlates with mental arithmetic task demand [73], suggesting functional cohesion. In contrast, in human spatial attention experiments, alpha and gamma have been shown to display differential temporal modulation relative to a task stimulus and, in addition, to encode distinct features of task predictability [74], suggesting an independent mode of action. Finally, investigations in humans and macaques have revealed that alpha waves phasically modulate gamma activity and suppress neuronal firing [75], arguing for a hierarchical relationship.

Our analyses of alpha and gamma show for the first time that they are differentially modulated throughout the day in freely behaving animals which can be interpreted as accordance with the findings of [74]. Linear correlation between alpha and gamma frequency bands displays the lowest values. And in PCA, their alignment is orthogonal, which emphasizes that they might convey complementary information beyond the cortex, and that this might be the case throughout the entire 24-hour day. The latter is supported by the fact that circadian sine-wave oscillations of alpha and gamma peak in antiphase in wild-type animals. As revealed by PCA, both alpha bands show the biggest contribution to variation in our data and substantially distinguish wild-type from *Cry1/2*^*−/−*^ animals with their decrease in linear correlation during DD. Accordingly, coherence measurements show that noticeable differences occur predominantly in the alpha bands (around 10 Hz), with increased coherence in *Cry1/2*^*−/−*^ animals during DD. Both gamma bands, on the other hand, show the highest sine-wave amplitudes, the lowest deviation from 24-hour periods in wild-type animals, and the lowest sample entropy values. Our sine wave analyses reveal that their individual circadian pattern is dependent on an intact endogenous circadian clock and environmental rhythms. Thus, we identify alpha and gamma frequency contents as promising targets for further dissection of spectrally directed connectivity [76], especially in the context of its circadian modulation.

In addition to the new insights gained from our study it raises questions how these complex dynamical activity changes of groups of neurons emerge from the molecular clock deficiency in *Cry1/2*^*−/−*^ animals and to what extent the changes in neuronal activity based on circadian disturbances might be related to the development of pathologies. Previous studies have described the importance of gap junctions in SCN neurons [77], coupling and synchronizing their electrical activity. It has also been shown that action potentials in SCN neurons contribute to maintaining population synchronicity [78]. As we recorded from the extracellular space, we can only make inferences about individual neurons’ activity. Based on the assumption that their synchronicity and the directedness of their activity shapes the LFP we recorded [58], one might still speculate about network properties within the SCN. In *Cry*^*−/−*^ animals, we saw increased LFP amplitude and abnormal rhythms at the same time. Here, the molecular machinery for cellular oscillations is abolished, so mutual dynamics between neurons to reinforce synchronicity and rhythmicity, as described for the intact SCN [79], cannot persist. The interceptive and corrective dynamics of individually firing SCN neurons in wild-type animals [80] pointing in different directions and partially cancelling each other out might cause their relatively lower LFP amplitude. Blunt coupling of impulsive neuronal activity without individual dynamics, on the other hand, leads to higher overall synchronicity on a population level, but less rhythmicity and regularity over 24-h timespans in *Cry1/2*^*−/−*^ animals. Finally, several independent studies could establish connections between expression of components of the TTL and the regulation of ion channels in the SCN [17], hinting towards possible explanatory frameworks for the increase of LFP amplitude observed in this study. Yet, it remains unclear how the individual elements of the molecular machinery synergistically give rise to circadian patterns of electrical activity in the SCN.

The NAc, on the other hand, has been found to mainly consist of neurons that do not generate spontaneous firing and that largely depend on inputs from other brain regions such as the cortex or limbic areas [81]. One study could establish a functional role for gap junctions in the NAc [82], however, it seems unlikely that they play a similar role as reported for the SCN. NAc neurons show rhythmic behavior, as further evidenced by our study, and also their gene and protein expression have been shown to be under circadian regulation [28, 31, 83]. Interestingly, in a different study *Clock*^*Δ19*^ mice were shown to display deficits in low-gamma cross-frequency phase coupling and neuronal phase locking in the NAc [84] and dysfunctional gamma oscillatory tuning was linked to reduced anxiety in these animals [85], further emphasizing the functional connection between the endogenous clock, neuronal activity, and behavior. While only insufficiently characterized indirect projections from the SCN to the NAc exist, the NAc seems to play an independent role in sleep-wake regulation [86]. Our experiments did not specifically test any functional organization of the two areas. Yet, the results from Granger causality analysis hint towards a hierarchical relationship between SCN and NAc, where SCN activity dictates broad activity levels in the NAc over the course of the day.

Studies on different neurophysiological systems have already shown that LFPs are not simple passive representations of a systems’ dynamical state, but can actively guide information processing [87, 88] and have explanatory value for neuropsychiatric conditions [76]. It is known that in humans both endogenous circadian characteristics, caused by polymorphisms of clock genes, and exogenous disturbances, such as shift work, often significantly increase the probability of developing, e.g., mental disorders [89–91]. Likewise, exogenous circadian perturbations in rodents can cause behavioral deficits resembling neuropsychiatric disorders [92–94]. Specifically related to our model, recent studies have shown that *Cry1/2*^*−/−*^ animals display anxiety-like behavior and restlessness [95, 96] as well as reduced alcohol preference with an increased motivation to obtain it [92]. Our present study now shows that in these mice, even when kept in LD, local neuronal activity and its frequency components are significantly affected in diurnal distribution of activity, circadian periods, amplitude, and phase. We have previously suggested that such changes may disrupt cross-communication between brain regions due to perturbation of temporal synchronization or separation of their activities and lead to impaired behavior and susceptibility to pathologies [97]. Taken together, the changes in circadian and non-circadian neuronal activity patterns, as shown here in the NAc, could, for example, contribute to the above-mentioned reward-related deficits of *Cry1/2*^*−/−*^ mice [92, 98, 99].

In summary, our study describes for the first time that integrity of both endogenous circadian and environmental diurnal rhythms is fundamental for electrical coordination of neuronal ensemble dynamics in the SCN and the NAc and that it affects a multitude of emergent properties, such as circadian rhythmicity, LFP amplitude as well as correlation, regularity, and entropy of their electrical activity. Interestingly, this involves not only the temporal coordination of neuronal activity over the time span of a day, but also in the range of hours, minutes, seconds, and milliseconds. It also involves properties of neuronal functions within a brain region, between brain regions, and even between individual animals in a group. Thus, from our point of view, the scope of how circadian disturbances influence brain activity and directly link to the development of neuropsychiatric disorders becomes clearer and more evident. Conversely, it also shows that chronotherapeutic measures aiming at exogenous stabilization of circadian rhythms can have positive effects of great magnitude on brain functionality.

## Supplementary information

Supplementary Material

## Data Availability

Further information and requests for resources should be directed to and will be fulfilled by the lead contact, Paul Volkmann (paul.volkmann@dpag.ox.ac.uk). The datasets generated and analyzed during the current study are available from the corresponding author on reasonable request.

## Code availability

The code generated in the current study as well as any additional information required to reanalyze the data reported in this paper are available from the lead contact upon request.

## References

[1] Hyman SE. Can neuroscience be integrated into the DSM-V? Nat Rev Neurosci. 2007;8:725–32. 17704814 10.1038/nrn2218

[2] Uhlhaas PJ, Singer W. Neural synchrony in brain disorders: relevance for cognitive dysfunctions and pathophysiology. Neuron. 2006;52:155–68. 17015233 10.1016/j.neuron.2006.09.020

[3] Patke A, Young MW, Axelrod S. Molecular mechanisms and physiological importance of circadian rhythms. Nat Rev Mol Cell Biol. 2020;21:67–84. 31768006 10.1038/s41580-019-0179-2

[4] Hardin PE, Hall JC, Rosbash M. Feedback of the Drosophila period gene product on circadian cycling of its messenger RNA levels. Nature. 1990;343:536–40. 2105471 10.1038/343536a0

[5] Konopka RJ, Benzer S. Clock mutants of *Drosophila melanogaster*. Proc Natl Acad Sci USA. 1971;68:2112–6. 5002428 10.1073/pnas.68.9.2112 PMC389363

[6] Stephan FK, Zucker I. Circadian rhythms in drinking behavior and locomotor activity of rats are eliminated by hypothalamic lesions. Proc Natl Acad Sci USA. 1972;69:1583–6. 4556464 10.1073/pnas.69.6.1583 PMC426753

[7] Zehring WA, Wheeler DA, Reddy P, Konopka RJ, Kyriacou CP, Rosbash M, et al. P-element transformation with period locus DNA restores rhythmicity to mutant, arrhythmic drosophila melanogaster. Cell. 1984;39:369–76. 6094014 10.1016/0092-8674(84)90015-1

[8] Hastings MH, Maywood ES, Brancaccio M. Generation of circadian rhythms in the suprachiasmatic nucleus. Nat Rev Neurosci. 2018;19:453–69. 29934559 10.1038/s41583-018-0026-z

[9] Libert S, Bonkowski MS, Pointer K, Pletcher SD, Guarente L. Deviation of innate circadian period from 24 h reduces longevity in mice: Impact of circadian clock on longevity. Aging Cell. 2012;11:794–800. 22702406 10.1111/j.1474-9726.2012.00846.x PMC3526942

[10] Mohawk JA, Green CB, Takahashi JS. Central and peripheral circadian clocks in mammals. Annu Rev Neurosci. 2012;35:445–62. 22483041 10.1146/annurev-neuro-060909-153128 PMC3710582

[11] Gekakis N, Staknis D, Nguyen HB, Davis FC, Wilsbacher LD, King DP, et al. Role of the CLOCK protein in the mammalian circadian mechanism. Science. 1998;280:1564–9. 9616112 10.1126/science.280.5369.1564

[12] Kume K, Zylka MJ, Sriram S, Shearman LP, Weaver DR, Jin X, et al. mCRY1 and mCRY2 are essential components of the negative limb of the circadian clock feedback loop. Cell. 1999;98:193–205. 10428031 10.1016/s0092-8674(00)81014-4

[13] Weaver DR. The suprachiasmatic nucleus: a 25-year retrospective. J Biol Rhythms. 1998;13:100–12. 9554572 10.1177/074873098128999952

[14] Farajnia S, Michel S, Deboer T, vanderLeest HT, Houben T, Rohling JHT, et al. Evidence for neuronal desynchrony in the aged suprachiasmatic nucleus clock. J Neurosci. 2012;32:5891–9. 22539850 10.1523/JNEUROSCI.0469-12.2012 PMC6703600

[15] Ralph MR, Foster RG, Davis FC, Menaker M. Transplanted suprachiasmatic nucleus determines circadian period. Science. 1990;247:975–8. 2305266 10.1126/science.2305266

[16] Im C, Seo J-M. A review of electrodes for the electrical brain signal recording. Biomed Eng Lett. 2016;6:104–12.

[17] Belle MD, Allen CN. The circadian clock: a tale of genetic–electrical interplay and synaptic integration. Curr Opin Physiol. 2018;5:75–79. 31011692 10.1016/j.cophys.2018.08.002 PMC6474415

[18] Iyer AR, Sheeba V. A new player in circadian networks: Role of electrical synapses in regulating functions of the circadian clock. Front Physiol. 2022;13:968574. 36406999 10.3389/fphys.2022.968574 PMC9669436

[19] Koronowski KB, Sassone-Corsi P. Communicating clocks shape circadian homeostasis. Science. 2021;371:eabd0951. 33574181 10.1126/science.abd0951 PMC8123919

[20] McCauley JP, Petroccione MA, D’Brant LY, Todd GC, Affinnih N, Wisnoski JJ, et al. Circadian modulation of neurons and astrocytes controls synaptic plasticity in hippocampal area CA1. Cell Rep. 2020;33:108255. 33053337 10.1016/j.celrep.2020.108255 PMC7700820

[21] Tokuda IT, Ono D, Honma S, Honma K-I, Herzel H. Coherency of circadian rhythms in the SCN is governed by the interplay of two coupling factors. PLoS Comput Biol. 2018;14:e1006607. 30532130 10.1371/journal.pcbi.1006607 PMC6301697

[22] Albus H, Bonnefont X, Chaves I, Yasui A, Doczy J, Van Der Horst GTJ, et al. Cryptochrome-deficient mice lack circadian electrical activity in the suprachiasmatic nuclei. Curr Biol. 2002;12:1130–3. 12121621 10.1016/s0960-9822(02)00923-5

[23] Katzner S, Nauhaus I, Benucci A, Bonin V, Ringach DL, Carandini M. Local origin of field potentials in visual cortex. Neuron. 2009;61:35–41. 19146811 10.1016/j.neuron.2008.11.016 PMC2730490

[24] Mitzdorf U. Current source-density method and application in cat cerebral cortex: investigation of evoked potentials and EEG phenomena. Physiol Rev. 1985;65:37–100. 3880898 10.1152/physrev.1985.65.1.37

[25] Liu D, Li J, Wu J, Dai J, Chen X, Huang Y, et al. Monochromatic blue light activates suprachiasmatic nucleus neuronal activity and promotes arousal in mice under sevoflurane anesthesia. Front Neural Circuits. 2020;14:55. 32973462 10.3389/fncir.2020.00055 PMC7461971

[26] Inouye ST, Kawamura H. Persistence of circadian rhythmicity in a mammalian hypothalamic ‘island’ containing the suprachiasmatic nucleus. Proc Natl Acad Sci USA. 1979;76:5962–6. 293695 10.1073/pnas.76.11.5962 PMC411773

[27] Frederick A, Bourget-Murray J, Chapman CA, Amir S, Courtemanche R. Diurnal influences on electrophysiological oscillations and coupling in the dorsal striatum and cerebellar cortex of the anesthetized rat. Front Syst Neurosci. 2014;8:145. 25309348 10.3389/fnsys.2014.00145 PMC4163932

[28] Landgraf D, Long JE, Welsh DK. Depression-like behaviour in mice is associated with disrupted circadian rhythms in nucleus accumbens and periaqueductal grey. Eur J Neurosci. 2016;43:1309–20. 26414405 10.1111/ejn.13085

[29] Huang X, Tao Q, Ren C. A comprehensive overview of the neural mechanisms of light therapy. Neurosci Bull. 2024;40:350–62. 37555919 10.1007/s12264-023-01089-8 PMC10912407

[30] Michel S, Nakamura TJ, Meijer JH, Colwell CS Electrophysiological approaches to studying the suprachiasmatic nucleus. In: Brown SA, editor. Circadian Clocks, vol. 2130, New York, NY: Springer US; 2021. p. 303-24. 10.1007/978-1-0716-0381-9_23 33284454

[31] Becker-Krail DD, Walker WH, Nelson RJ. The ventral tegmental area and nucleus accumbens as circadian oscillators: implications for drug abuse and substance use disorders. Front Physiol. 2022;13:886704. 35574492 10.3389/fphys.2022.886704 PMC9094703

[32] Papazoglou A, Lundt A, Wormuth C, Ehninger D, Henseler C, Soós J, et al. Non-restraining EEG Radiotelemetry: Epidural and Deep Intracerebral Stereotaxic EEG Electrode Placement. JoVE. 2016:54216. 10.3791/54216 PMC4993274 27404845

[33] Fernandez LMJ, Comte J-C, Le Merre P, Lin J-S, Salin P-A, Crochet S Highly Dynamic Spatiotemporal Organization of Low-Frequency Activities During Behavioral States in the Mouse Cerebral Cortex. Cereb Cortex. 2016:cercor;bhw311v1. 10.1093/cercor/bhw311 27742711

[34] Soltani S, Chauvette S, Bukhtiyarova O, Lina J-M, Dubé J, Seigneur J, et al. Sleep–wake cycle in young and older mice. Front Syst Neurosci. 2019;13:51. 31611779 10.3389/fnsys.2019.00051 PMC6769075

[35] Weiergräber M, Henry M, Hescheler J, Smyth N, Schneider T. Electrocorticographic and deep intracerebral EEG recording in mice using a telemetry system. Brain Res Protoc. 2005;14:154–64. 10.1016/j.brainresprot.2004.12.006 15795169

[36] Lundt A, Wormuth C, Siwek ME, Müller R, Ehninger D, Henseler C, et al. EEG radiotelemetry in small laboratory rodents: a powerful state-of-the art approach in neuropsychiatric, neurodegenerative, and epilepsy research. Neural Plasticity. 2016;2016:1–19. 10.1155/2016/8213878 PMC4706962 26819775

[37] Paxinos G, Franklin KBJ Paxinos and Franklin’s The mouse brain in stereotaxic coordinates. Fifth edition. London: Academic Press, an imprint of Elsevier; 2019.

[38] Heideman M, Johnson D, Burrus C. Gauss and the history of the fast fourier transform. IEEE ASSP Mag. 1984;1:14–21.

[39] R Core Team. R: A language and environment for statistical computing. R Foundation for Statistical Computing, Vienna, Austria. https://www.R-project.org. 2022.

[40] Markowski CA, Markowski EP. Conditions for the effectiveness of a preliminary test of variance. Am Statistician. 1990;44:322–6.

[41] Wickham H. ggplot2: Elegant Graphics for Data Analysis. Springer-Verlag New York. ISBN 978-3-319-24277-4. 2016. https://ggplot2.tidyverse.org.

[42] Kolde R pheatmap: Pretty Heatmaps. R package version 1.0.12. 2019. https://github.com/raivokolde/pheatmap.

[43] signal developers. signal: Signal processing. 2023. https://r-forge.r-project.org/projects/signal.

[44] Torrence C, Compo GP. A practical guide to wavelet analysis. Bull Am Meteor Soc. 1998;79:61–78.

[45] Greenblatt RE, Pflieger ME, Ossadtchi AE. Connectivity measures applied to human brain electrophysiological data. J Neurosci Methods. 2012;207:1–16. 22426415 10.1016/j.jneumeth.2012.02.025 PMC5549799

[46] Karvat G, Schneider A, Alyahyay M, Steenbergen F, Tangermann M, Diester I. Real-time detection of neural oscillation bursts allows behaviourally relevant neurofeedback. Commun Biol. 2020;3:72. 32060396 10.1038/s42003-020-0801-z PMC7021904

[47] Gouhier TC, Grinsted A, Simko V R package biwavelet: Conduct Univariate and Bivariate Wavelet Analyses. (Version 0.20.21), 2021. https://github.com/tgouhier/biwavelet.

[48] Zeileis A, Grothendieck G zoo: S3 Infrastructure for Regular and Irregular Time Series. J Stat Soft. 2005;14.

[49] Ruf T. The lomb-scargle periodogram in biological rhythm research: analysis of incomplete and unequally spaced time-series. Biol Rhythm Res. 1999;30:178–201. 10.1076/brhm.30.2.149.1424 11708361

[50] Trapletti A, Hornik K tseries: Time Series Analysis and Computational Finance. R package version 0.10-43. 2023.

[51] Sueur J, Aubin T, Simonis C. Seewave, a free modular tool for sound analysis and synthesis. Bi oacoustics. 2008;18:213–26.

[52] Komsta L, Novomestky F moments: Moments, Cumulants, Skewness, Kurtosis and Related Tests. 2022. http://www.komsta.net.

[53] Richman JS, Moorman JR. Physiological time-series analysis using approximate entropy and sample entropy. Am J Physiol-Heart Circulatory Physiol. 2000;278:H2039–H2049. 10.1152/ajpheart.2000.278.6.H2039 10843903

[54] Tomcala J TSEntropies: Time Series Entropies. R package version 0.9, 2018. https://CRAN.R-project.org/package=TSEntropies.

[55] Zeileis A, Hothorn T Diagnostic Checking in Regression Relationships. 2002.

[56] Vu V ggbiplot: A ggplot2 based biplot. https://github.com/vqv/ggbiplot. 2011.

[57] Fransen AMM, Van Ede F, Maris E. Identifying neuronal oscillations using rhythmicity. NeuroImage. 2015;118:256–67. 26054877 10.1016/j.neuroimage.2015.06.003

[58] Buzsáki G, Anastassiou CA, Koch C. The origin of extracellular fields and currents-EEG, ECoG, LFP and spikes. Nat Rev Neurosci. 2012;13:407–20. 22595786 10.1038/nrn3241 PMC4907333

[59] Gallego-Carracedo C, Perich MG, Chowdhury RH, Miller LE, Gallego JÁ. Local field potentials reflect cortical population dynamics in a region-specific and frequency-dependent manner. eLife. 2022;11:e73155. 35968845 10.7554/eLife.73155 PMC9470163

[60] Baldauf D, Desimone R. Neural mechanisms of object-based attention. Science. 2014;344:424–7. 24763592 10.1126/science.1247003

[61] Meador KJ, Ray PG, Echauz JR, Loring DW, Vachtsevanos GJ. Gamma coherence and conscious perception. Neurology. 2002;59:847–54. 12297565 10.1212/wnl.59.6.847

[62] Turner JR, Wit M, Hajos T, Wit M, Howren MB, Insana S, et al. Quantitative EEG Including the Five Common Bandwidths (Delta, Theta, Alpha, Sigma, and Beta). In: Gellman MD, Turner JR, editors. Encyclopedia of Behavioral Medicine, New York, NY: Springer New York; 2013. p. 1606-9.

[63] Van Dongen HP, Olofsen E, VanHartevelt JH, Kruyt EW. Searching for biological rhythms: peak detection in the periodogram of unequally spaced data. J Biol Rhythms. 1999;14:617–20. 10643760 10.1177/074873099129000984

[64] Stevner ABA, Vidaurre D, Cabral J, Rapuano K, Nielsen SFV, Tagliazucchi E, et al. Discovery of key whole-brain transitions and dynamics during human wakefulness and non-REM sleep. Nat Commun. 2019;10:1035. 30833560 10.1038/s41467-019-08934-3 PMC6399232

[65] Lima B, Singer W, Chen N-H, Neuenschwander S. Synchronization dynamics in response to plaid stimuli in monkey V1. Cereb Cortex. 2010;20:1556–73. 19812238 10.1093/cercor/bhp218 PMC2882822

[66] Groth D, Hartmann S, Klie S, Selbig J Principal Components Analysis. In: Reisfeld B, Mayeno AN, editors. Computational Toxicology, 930, Totowa, NJ: Humana Press; 2013. p. 527-47. 10.1007/978-1-62703-059-5_22 23086856

[67] Shirakawa T, Honma S, Katsuno Y, Oguchi H, Honma K. Synchronization of circadian firing rhythms in cultured rat suprachiasmatic neurons: Synchronization of neuronal rhythms in the SCN. Eur J Neurosci. 2000;12:2833–8. 10971625 10.1046/j.1460-9568.2000.00170.x

[68] Moore RY, Speh JC. GABA is the principal neurotransmitter of the circadian system. Neurosci Lett. 1993;150:112–6. 8097023 10.1016/0304-3940(93)90120-a

[69] Seguin C, Sporns O, Zalesky A. Brain network communication: concepts, models and applications. Nat Rev Neurosci. 2023;24:557–74. 37438433 10.1038/s41583-023-00718-5

[70] Mesgar S, Eskandari K, Karimian-Sani-Varjovi H, Salemi-Mokri-Boukani P, Haghparast A. The dopaminergic system modulates the electrophysiological activity of the suprachiasmatic nucleus dependent on the circadian cycle. Neurochem Res. 2023;48:3420–9. 37452257 10.1007/s11064-023-03988-8

[71] Buzsáki G, Draguhn A. Neuronal oscillations in cortical networks. Science. 2004;304:1926–9. 15218136 10.1126/science.1099745

[72] Donnelly NA, Holtzman T, Rich PD, Nevado-Holgado AJ, Fernando ABP, Van Dijck G, et al. Oscillatory activity in the medial prefrontal cortex and nucleus accumbens correlates with impulsivity and reward outcome. PLoS ONE. 2014;9:e111300. 25333512 10.1371/journal.pone.0111300 PMC4205097

[73] Palva JM, Palva S, Kaila K. Phase synchrony among neuronal oscillations in the human cortex. J Neurosci. 2005;25:3962–72. 15829648 10.1523/JNEUROSCI.4250-04.2005 PMC6724920

[74] Bauer M, Stenner M-P, Friston KJ, Dolan RJ. Attentional modulation of alpha/beta and gamma oscillations reflect functionally distinct processes. J Neurosci. 2014;34:16117–25. 25429152 10.1523/JNEUROSCI.3474-13.2014 PMC4244475

[75] Jensen O. Distractor inhibition by alpha oscillations is controlled by an indirect mechanism governed by goal-relevant information. Commun Psychol. 2024;2:36. 38665356 10.1038/s44271-024-00081-w PMC11041682

[76] Friston KJ, Bastos AM, Pinotsis D, Litvak V. LFP and oscillations—what do they tell us? Curr Opin Neurobiol. 2015;31:1–6. 25079053 10.1016/j.conb.2014.05.004 PMC4376394

[77] Colwell CS. Rhythmic coupling among cells in the suprachiasmatic nucleus. J Neurobiol. 2000;43:379–88. 10861563 10.1002/1097-4695(20000615)43:4<379::aid-neu6>3.0.co;2-0 PMC2577317

[78] Yamaguchi S, Isejima H, Matsuo T, Okura R, Yagita K, Kobayashi M, et al. Synchronization of cellular clocks in the suprachiasmatic nucleus. Science. 2003;302:1408–12. 14631044 10.1126/science.1089287

[79] Welsh DK, Takahashi JS, Kay SA. Suprachiasmatic nucleus: cell autonomy and network properties. Annu Rev Physiol. 2010;72:551–77. 20148688 10.1146/annurev-physiol-021909-135919 PMC3758475

[80] Welsh DK, Logothetis DE, Meister M, Reppert SM. Individual neurons dissociated from rat suprachiasmatic nucleus express independently phased circadian firing rhythms. Neuron. 1995;14:697–706. 7718233 10.1016/0896-6273(95)90214-7

[81] Floresco SB. The nucleus accumbens: an interface between cognition, emotion, and action. Annu Rev Psychol. 2015;66:25–52. 25251489 10.1146/annurev-psych-010213-115159

[82] Kokarovtseva L, Jaciw-Zurakiwsky T, Mendizabal Arbocco R, Frantseva MV, Perez Velazquez JL. Excitability and gap junction–mediated mechanisms in nucleus accumbens regulate self-stimulation reward in rats. Neuroscience. 2009;159:1257–63. 19409225 10.1016/j.neuroscience.2009.01.065

[83] DePoy LM, Petersen KA, Zong W, Ketchesin KD, Matthaei RC, Yin R, et al. Cell-type and sex-specific rhythmic gene expression in the nucleus accumbens. Mol Psychiatry. 2024. 10.1038/s41380-024-02569-7. PMC11449664 38678086

[84] Dzirasa K, Coque L, Sidor MM, Kumar S, Dancy EA, Takahashi JS, et al. Lithium ameliorates nucleus accumbens phase-signaling dysfunction in a genetic mouse model of mania. J Neurosci. 2010;30:16314–23. 21123577 10.1523/JNEUROSCI.4289-10.2010 PMC3165036

[85] Dzirasa K, McGarity DL, Bhattacharya A, Kumar S, Takahashi JS, Dunson D, et al. Impaired Limbic Gamma Oscillatory Synchrony during Anxiety-Related Behavior in a Genetic Mouse Model of Bipolar Mania. J Neurosci. 2011;31:6449–56. 21525286 10.1523/JNEUROSCI.6144-10.2011 PMC3112006

[86] Oishi Y, Xu Q, Wang L, Zhang B-J, Takahashi K, Takata Y, et al. Slow-wave sleep is controlled by a subset of nucleus accumbens core neurons in mice. Nat Commun. 2017;8:734. 28963505 10.1038/s41467-017-00781-4 PMC5622037

[87] Kayser C, Montemurro MA, Logothetis NK, Panzeri S. Spike-phase coding boosts and stabilizes information carried by spatial and temporal spike patterns. Neuron. 2009;61:597–608. 19249279 10.1016/j.neuron.2009.01.008

[88] Likhtik E, Stujenske JM, A Topiwala M, Harris AZ, Gordon JA. Prefrontal entrainment of amygdala activity signals safety in learned fear and innate anxiety. Nat Neurosci. 2014;17:106–13. 24241397 10.1038/nn.3582 PMC4035371

[89] Boivin DB, Boudreau P, Kosmadopoulos A. Disturbance of the circadian system in shift work and its health impact. J Biol Rhythms. 2022;37:3–28. 34969316 10.1177/07487304211064218 PMC8832572

[90] Jagannath A, Taylor L, Wakaf Z, Vasudevan SR, Foster RG. The genetics of circadian rhythms, sleep and health. Hum Mol Genet. 2017;26:R128–R138. 28977444 10.1093/hmg/ddx240 PMC5886477

[91] Walker WH, Walton JC, DeVries AC, Nelson RJ. Circadian rhythm disruption and mental health. Transl Psychiatry. 2020;10:28. 32066704 10.1038/s41398-020-0694-0 PMC7026420

[92] Hühne A, Echtler L, Kling C, Stephan M, Schmidt MV, Rossner MJ, et al. Circadian gene × environment perturbations influence alcohol drinking in Cryptochrome-deficient mice. Addict Biol. 2022;27:e13105. 34672045 10.1111/adb.13105

[93] Leach G, Adidharma W, Yan L. Depression-like responses induced by daytime light deficiency in the diurnal grass rat (Arvicanthis niloticus). PLoS ONE. 2013;8:e57115. 23437327 10.1371/journal.pone.0057115 PMC3577787

[94] Monje FJ, Cabatic M, Divisch I, Kim E-J, Herkner KR, Binder BR, et al. Constant Darkness Induces IL-6-Dependent Depression-Like Behavior through the NF- B Signaling Pathway. J Neurosci. 2011;31:9075–83. 21697358 10.1523/JNEUROSCI.1537-11.2011 PMC6623479

[95] Hühne A, Volkmann P, Stephan M, Rossner M, Landgraf D An in‐depth neurobehavioral characterization shows anxiety‐like traits, impaired habituation behavior, and restlessness in male *Cryptochrome* ‐deficient mice. Genes, Brain and Behavior. 2020. May. 10.1111/gbb.12661. 32348614

[96] De Bundel D, Gangarossa G, Biever A, Bonnefont X, Valjent E Cognitive dysfunction, elevated anxiety, and reduced cocaine response in circadian clock-deficient cryptochrome knockout mice. Front Behav Neurosci. 2013;7. 10.3389/fnbeh.2013.00152 PMC3807562 24187535

[97] Hühne A, Welsh DK, Landgraf D. Prospects for circadian treatment of mood disorders. Ann Med. 2018;50:637–54. 30265156 10.1080/07853890.2018.1530449

[98] Bayassi-Jakowicka M, Lietzau G, Czuba E, Patrone C, Kowiański P. More than addiction—the nucleus accumbens contribution to development of mental disorders and neurodegenerative diseases. IJMS. 2022;23:2618. 35269761 10.3390/ijms23052618 PMC8910774

[99] Grueter BA, Rothwell PE, Malenka RC. Integrating synaptic plasticity and striatal circuit function in addiction. Curr Opin Neurobiol. 2012;22:545–51. 22000687 10.1016/j.conb.2011.09.009 PMC3276730

