# Supplementary material for: Integrity of the circadian clock determines regularity of high-frequency and diurnal LFP rhythms within and between brain areas
Source: Mol Psychiatry. 2024 Oct 29;30(5):1859–75. doi: 10.1038/s41380-024-02795-z (PMC12015176; doi:10.1038/s41380-024-02795-z)
Supplement: Supplementary file 1 — Supplementary Material [file 41380_2024_2795_MOESM1_ESM.pdf]

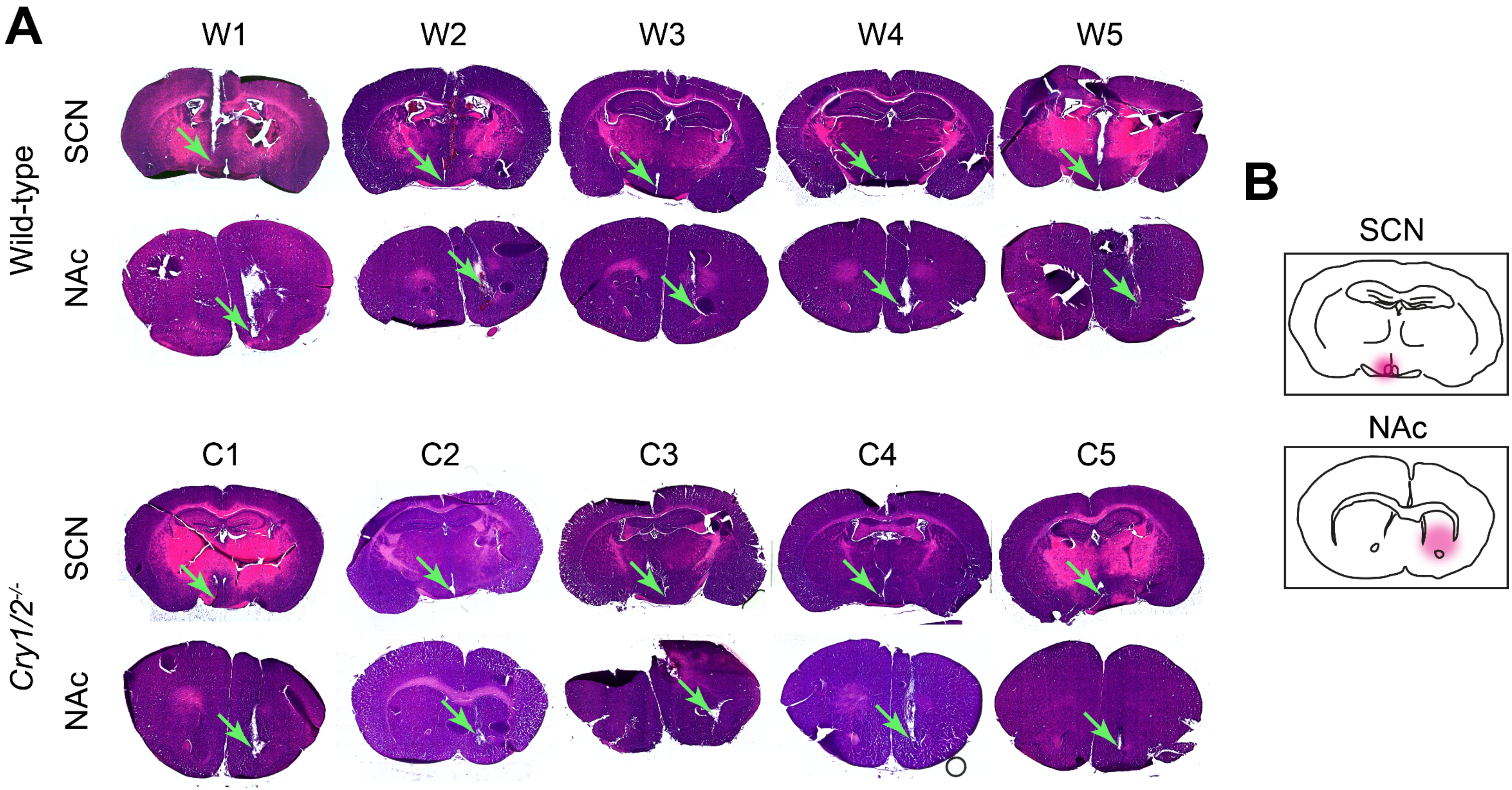

Supplementary Figure 1

(A) Images of hematoxylin-eosin-stained SCN and NAc slices for all five wild-type mice (W1-W5) and all five *Cry1/2*$^{-/-}$ mice (C1-C5) used in the study. The green arrow points at the position of the tip of the implanted electrode. (B) Schematic outlines of the brain planes in which the SCN and NAc are localized. The magenta cloud marks the area that was targeted for the electrode tip in the SCN and NAc, respectively.

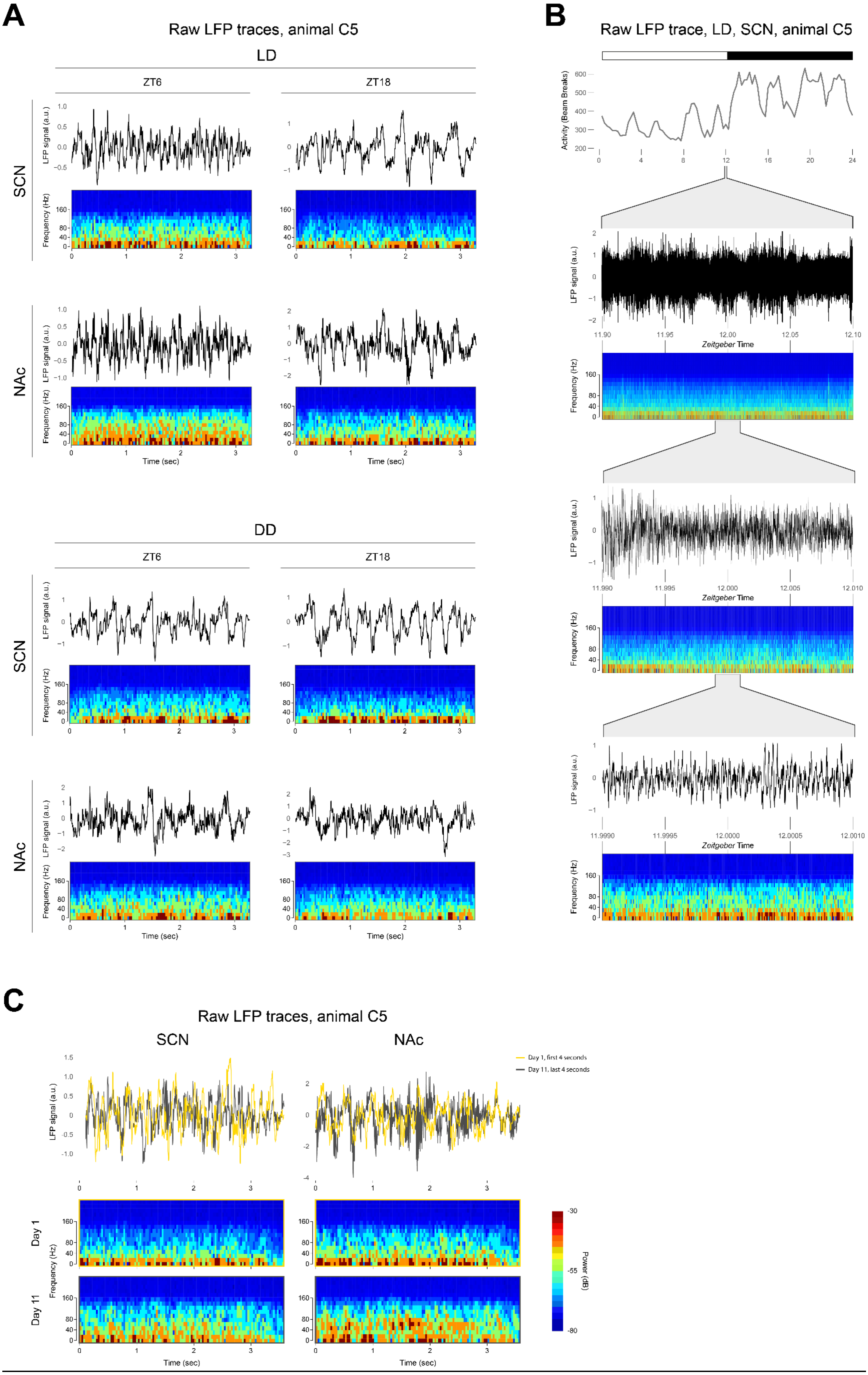

A
Raw LFP traces, animal C5
LD
ZT6
ZT18
SCN
NAc
DD
LFP signal (a.u.)
Frequency (Hz)
Time (sec)
B
Raw LFP trace, LD, SCN, animal C5
Activity (Beam Breaks)
Zeitgeber Time
C
SCN
NAc
Day 1, first 4 seconds
Day 11, last 4 seconds
Day 1
Day 11
Power (dB)

Supplementary Figure 2

(A) Raw LFP traces of representative animal C5 under LD and DD with corresponding spectrograms. Depicted are the first 3.6 seconds at ZT6 and ZT18 in the SCN and NAc of the recording period used for all further analyses in this paper. Color scale as in (C). (B) Raw LFP traces of representative animal C5 under LD in the SCN. To show that the data is not contaminated by movement artifacts, we chose a segment around ZT12 when the animal's locomotor activity steeply increases (top). Shown below are raw LFP traces and the corresponding spectrogram with increasing time resolution spanning 360, 36, and 3.6 seconds around ZT12. Color scale as in (C). (C) Raw LFP traces of representative animal C5 from the beginning of the recording (day 1, ZT0-0.0015) overlayed with the end of recording (day 11, ZT23.9985-24) for both SCN and NAc with corresponding spectrograms for day 1 and day 11 show that amplitude of the recorded signal was stable over the entire recording period. White and black bars in B represent light and dark, respectively, with white-black bars indicating data for LD and black bars for DD. a.u. = arbitrary unit.

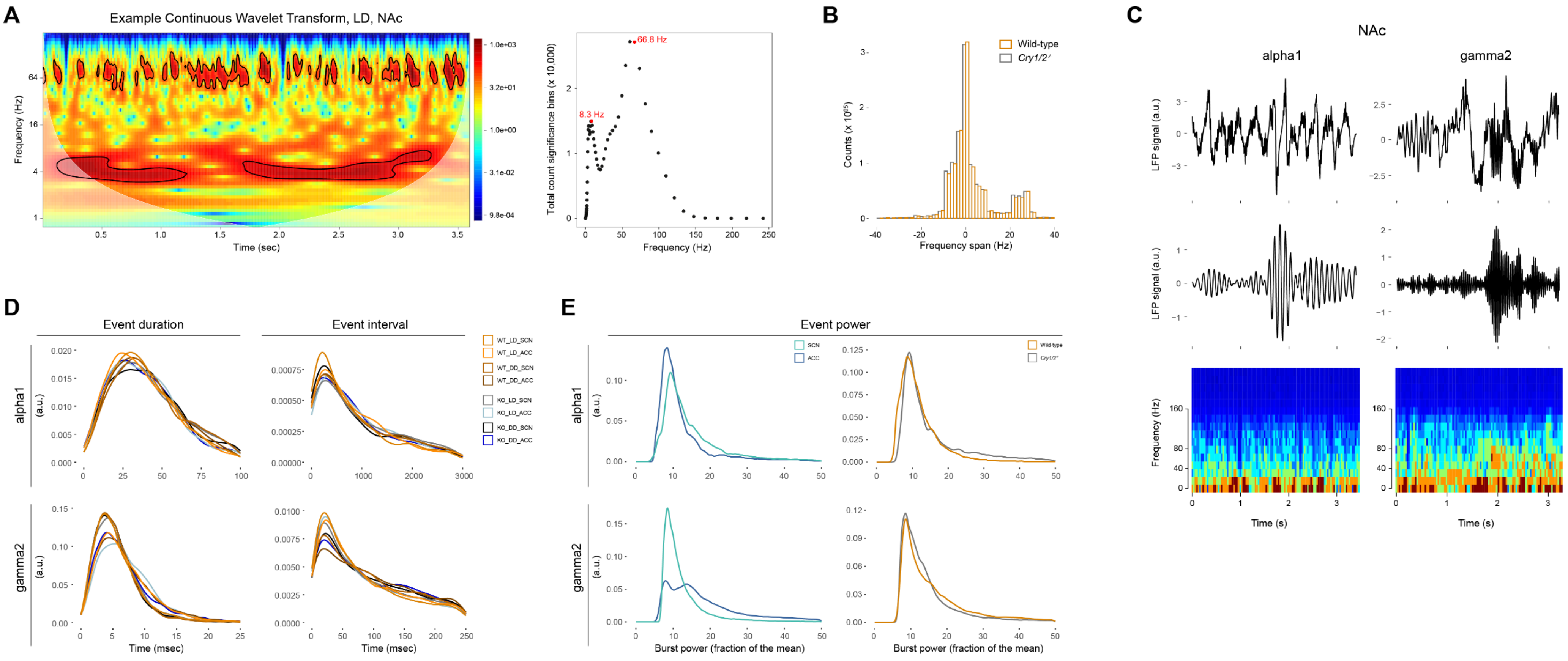

Supplementary Figure 3

(A) Time-frequency plane of a continuous wavelet transformation using Morlet wavelets of the NAc of representative animal C5 in LD from ZT0-0.001 (≙ 3.6 seconds). Color intensities show significance levels. Black borders highlight areas with a significance level above 1.96, the white-shaded area at the bottom on both ends marks the cone of influence (lower confidence for wavelets with longer periods towards the edges). The point plot on the right summarizes the count of high-significance-level bouts of all animals in both areas and light regimens during the investigated 6-minute bouts at the four different *Zeitgeber* times (ZT0, 6, 12, and 18) per frequency. It highlights the two maxima centered around 8.3 and 66.8 Hz chosen for further analyses of oscillations in Fig. S3B-D. (B) Histogram showing frequency span of oscillatory bursts, compiled for both alpha1 and gamma2. Bursts were defined by events exceeding the 98th percentile of their respective frequency. Spread was assessed by counting consecutive neighboring frequency bouts meeting the same criterion. (C) Example 3.6 seconds raw LFP traces of representative animal C1 under LD with corresponding spectrograms at the bottom to display alpha1 (left, ZT10.0255) and gamma2 (right, ZT2.8835) oscillations in the NAc. The top trace is the raw signal, the middle trace filtered at 8-11 Hz (alpha1) or 48-70 Hz (gamma2), respectively. (D) Density plots for burst duration (left) and burst interval of oscillatory events (right), each shown for gamma2 (top) and alpha1 (bottom). (E) Density plots of burst power

as fraction of the median (FOM). The left plots show remarkable differences between SCN and NAc depending on frequency, the right plots show differences by genotype (top: gamma2, bottom: alpha1)

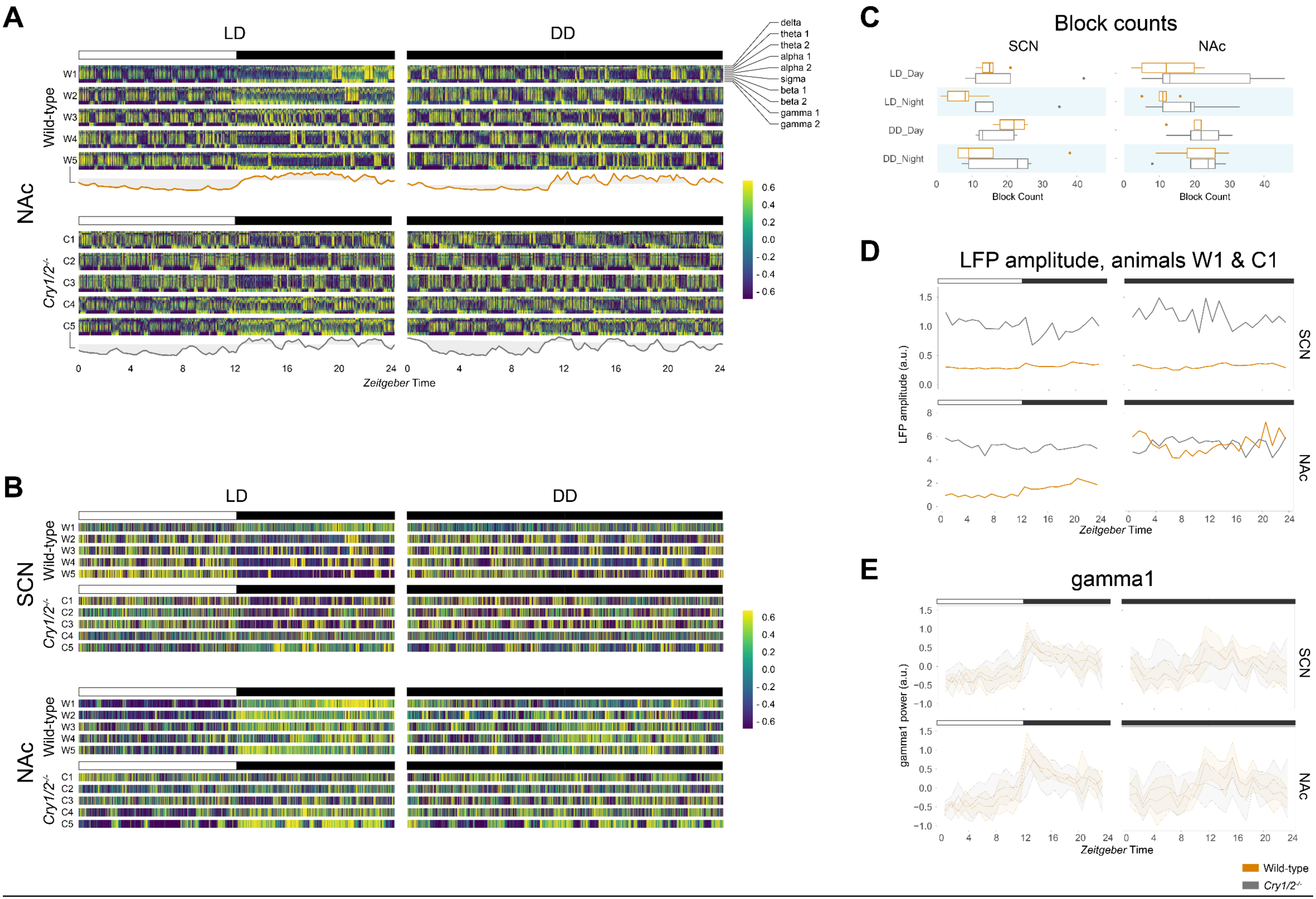

A
LD
DD
NAc
Wild-type
W1
W2
W3
W4
W5
Cry1/2-/-
C1
C2
C3
C4
C5
delta
theta 1
theta 2
alpha 1
alpha 2
sigma
beta 1
beta 2
gamma 1
gamma 2
0.6
0.4
0.2
0.0
- 0.2
- 0.4
- 0.6
0 4 8 12 16 20 24
Zeitgeber Time
B
SCN
C
Block counts
LD_Day
LD_Night
DD_Day
DD_Night
Block Count
D
LFP amplitude, animals W1 & C1
LFP amplitude (a.u.)
E
gamma1
gamma1 power (a.u.)
Wild-type
Cry1/2-/-

Supplementary Figure 4

(A) Individual spectrograms of 24-hour NAc activity of all five wild-type animals W1-W5 and all five *Cry1/2*$^{-/-}$ animals C1-C5 under LD and DD. Each frequency band was z-scored individually. The color gradient from purple to yellow describes values from minimum 0.75 standard deviations below the mean to maximum 0.75 standard deviations above the mean, respectively. Below the spectrograms of animals W5 and C5, their corresponding locomotor activity levels are shown. The shading in this curve represents activity levels below and above the average activity during the 24-h period. (B) Individual 24-h z-scored LFP fast Fourier transformed amplitude series from SCN and NAc under LD and DD of all five wild-type animals W1-W5 and all five *Cry1/2*$^{-/-}$ animals C1-C5. The color gradient from purple to yellow describes values from minimum 0.75 standard deviations below the mean to maximum 0.75 standard deviations above the mean, respectively. (C) Comparison of number of blocks counted from block time series (not depicted) of the rolling average calculated for each LFP time series. Data are plotted as boxplots with median and whiskers indicating 1.5-fold distance of inter-quartile range (IQR) to the upper/lower quartile. A Type II ANOVA showed a significant effect only for genotype (see Suppl. Table). (D) 24-h time series of LFP fast Fourier transformed amplitude of the SCN and the NAc within one-hour bins for single representative animals of each genotype (W1 and C1). (E) Gamma1 activity with mean ± standard deviation for one-hour bins of individual time series for each genotype for SCN (top panels) and NAc (bottom panels) under LD (left) and DD (right). White and black bars in A, B, D, and E represent light and dark, respectively, with white-black bars indicating data for LD and black bars for DD. a.u. = arbitrary unit.

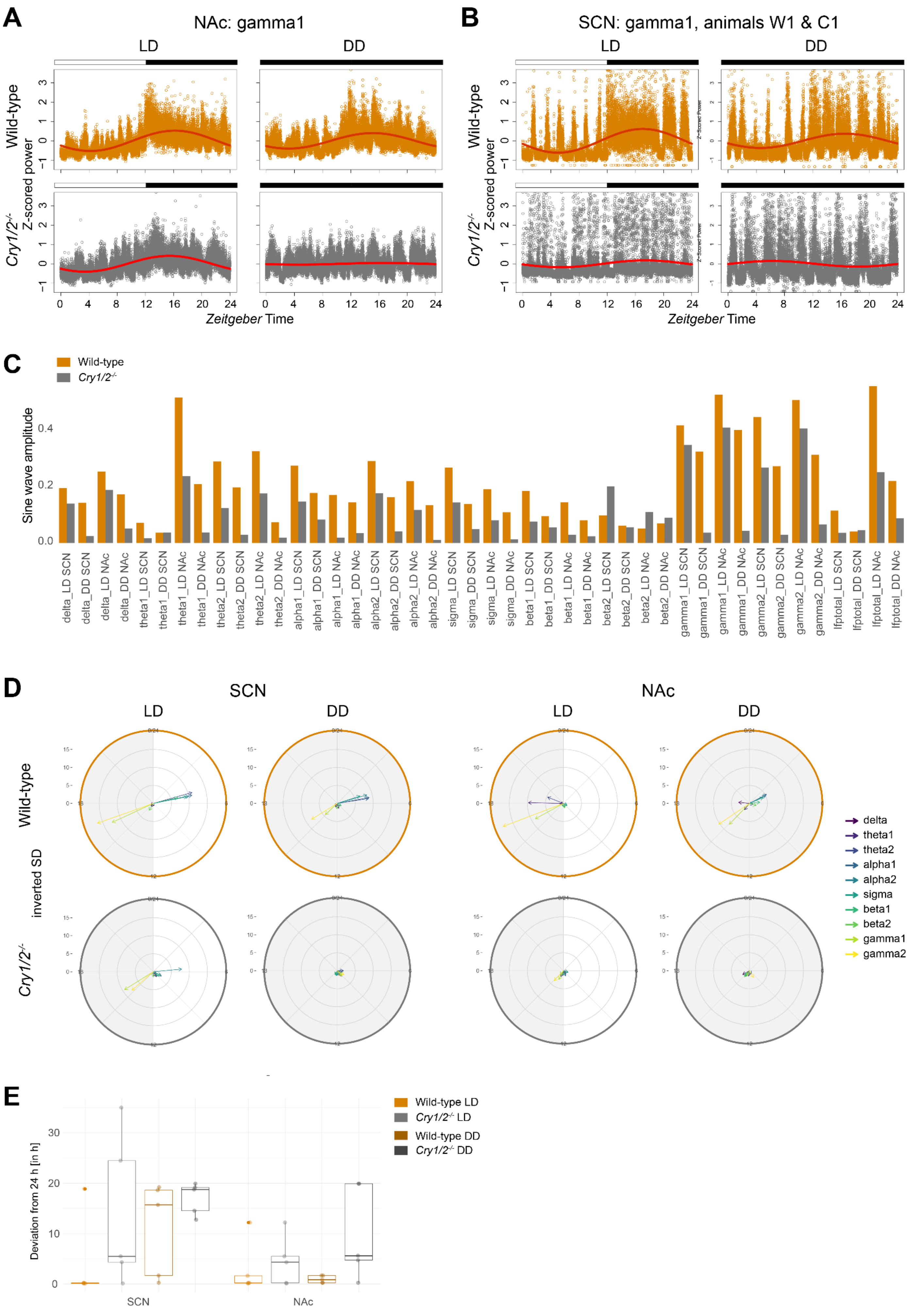
A
NAc: gamma1
LD
DD
Wild-type
Cry1/2-/-
Z-scored power
Zeitgeber Time
B
SCN: gamma1, animals W1 & C1
LD
DD
Wild-type
Cry1/2-/-
Z-scored power
Zeitgeber Time
C
Wild-type
Cry1/2-/-
Sine wave amplitude
D
SCN
NAc
LD
DD
LD
DD
Wild-type
inverted SD
Cry1/2-/-
delta
theta1
theta2
alpha1
alpha2
sigma
beta1
beta2
gamma1
gamma2
E
Wild-type LD
Cry1/2-/- LD
Wild-type DD
Cry1/2-/- DD
Deviation from 24 h [in h]
SCN
NAc

Supplementary Figure 5

(A) Linear sine wave fit for pooled z-scored gamma1 activity of all animals for each genotype in LD and DD in the NAc. (B) Linear sine wave fit for z-scored 24-h gamma1 SCN activity of single representative animals W1 and C1. (C) Comparison of wild-type and *Cry1/2*$^{-/-}$ sine wave amplitudes of pooled z-scored data for every frequency - light regimen - brain area pair. (D) 24-hour circular graphs with arrows pointing at the *Zeitgeber* time at which the highest amplitude of the fitted sine wave of all individual frequencies occurred. ZT timing of sine waves was calculated for individual animals and then averaged for each genotype in each light regimen - brain area pair. Lengths of arrows indicate the inverted standard deviation across animals within the respective frequency. (E) Genotype differences in absolute deviation of Lomb-Scargle peaks from 24 hours of LFP time series under different light regimens, separated by brain area and plotted as boxplots with median and whiskers indicating 1.5-fold distance of IQR to the upper/lower quartile. A Type II ANOVA showed a significant effect for genotype and brain area (see Suppl. Table). White and black bars in A represent light and dark, respectively, with white-black bars indicating data for LD and black bars for DD.

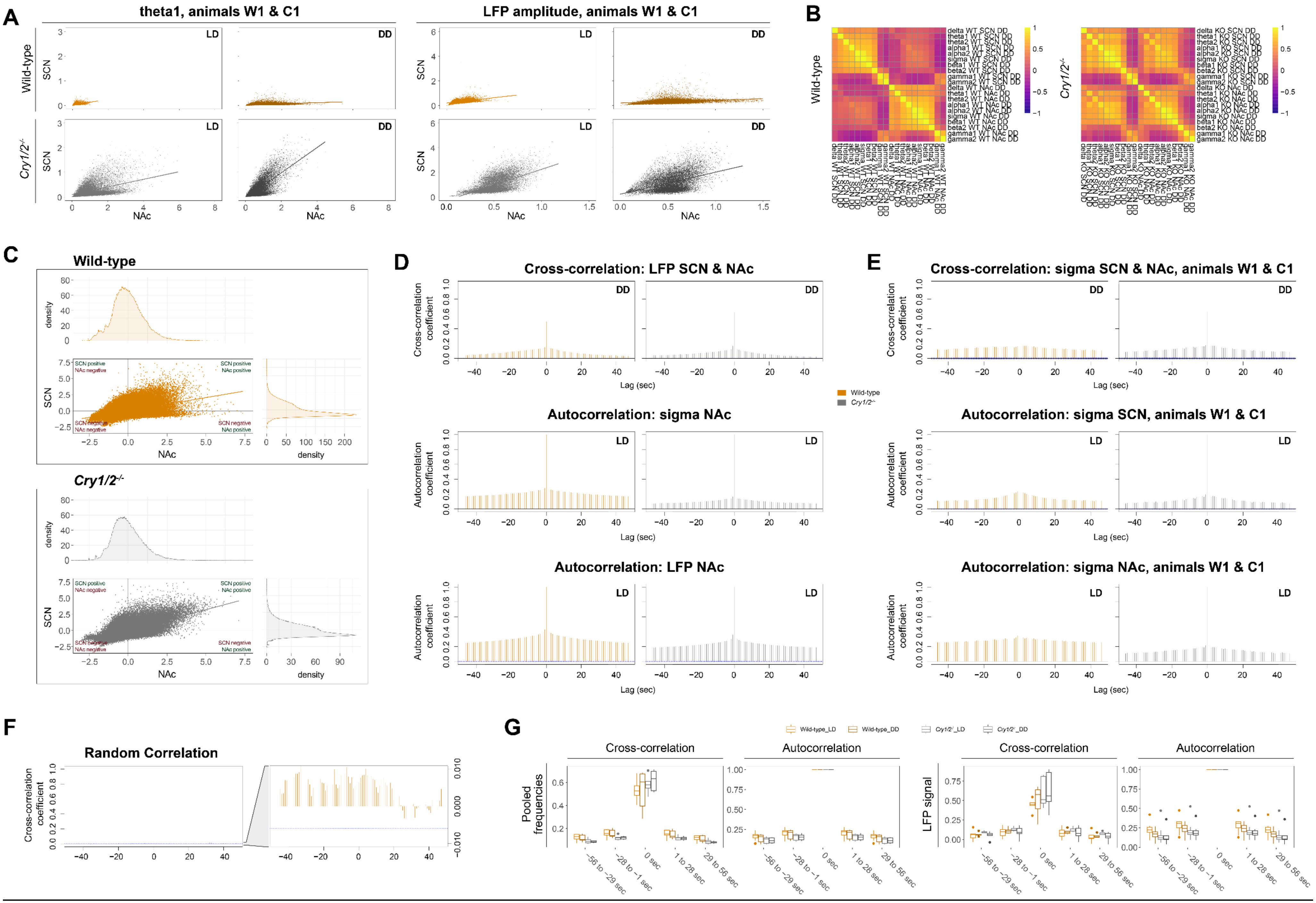
A
theta1, animals W1 & C1
LFP amplitude, animals W1 & C1
Wild-type
Cry1/2-/-
SCN
NAc
LD
DD
B
Wild-type
Cry1/2-/-
C
Wild-type
Cry1/2-/-
density
SCN
NAc
SCN positive
NAc negative
SCN positive
NAc positive
SCN negative
NAc negative
SCN negative
NAc positive
D
Cross-correlation: LFP SCN & NAc
Autocorrelation: sigma NAc
Autocorrelation: LFP NAc
Cross-correlation coefficient
Autocorrelation coefficient
Lag (sec)
Wild-type
Cry1/2-/-
E
Cross-correlation: sigma SCN & NAc, animals W1 & C1
Autocorrelation: sigma SCN, animals W1 & C1
Autocorrelation: sigma NAc, animals W1 & C1
F
Random Correlation
Cross-correlation coefficient
G
Wild-type_LD
Wild-type_DD
Cry1/2-/-_LD
Cry1/2-/-_DD
Cross-correlation
Autocorrelation
Pooled frequencies
LFP signal
-56 to -29 sec
-28 to -1 sec
0 sec
1 to 28 sec
29 to 56 sec

Supplementary Figure 6

(A) Pearson correlation of theta1 activity and LFP amplitude from representative animals of both genotypes (W1 and C1). (B) Pearson correlation color matrix for all frequency pairs from pooled data of each genotype for LD. Data is shown for the entire 24-h day. The color scale refers to correlation values from -1 (blue) to 1 (yellow) without truncation. (C) Pearson correlation of pooled z-scored theta1 activity between NAc and SCN for wild-type (top) and *Cry1/2*$^{-/-}$ animals (bottom) under LD with density plots for point clouds. The plot comprises pooled data from ZT0-24 while data shown in Fig. 3D is binned in 3-hour bins. The displayed data, unlike in Fig. 3A, 3B and S6A, has been z-scored. The black horizontal (y=0) and vertical (x=0) lines divide data into four quadrants that are used for the count plot in Fig. 3D: The horizontal line (y=0) divides data into high (above) and low (below) SCN activity relative to the mean; the vertical line (x=0) divides it into low (left) and high (right) NAc activity relative to the mean. (D) Top: cross-correlation of LFP amplitude between SCN and NAc under DD. Middle: autocorrelation of sigma1 activity in the NAc under LD. Bottom: autocorrelation of LFP amplitude in the NAc under LD. Each bar refers to a correlation value with a multiple of a 1-second time-lag. Values on the x-axis signify the lag in seconds for the corresponding correlation value. (E) Single representative animals W1 and C1. Top: cross-correlation of sigma activity between SCN and NAc under LD. Middle: autocorrelation of sigma activity in the SCN under LD. Bottom: autocorrelation of sigma activity in the NAc under LD. Depiction as in D. (F) Cross-correlation of LFP activity in the SCN under LD with activity in the NAc under DD (negative control for cross-correlation analysis). Depiction as in D. (G) Quantification of average correlation values in two different time lag windows with negative and positive lag each and at the same instance (middle boxplots). Left: all frequency bands pooled, right: LFP signal.

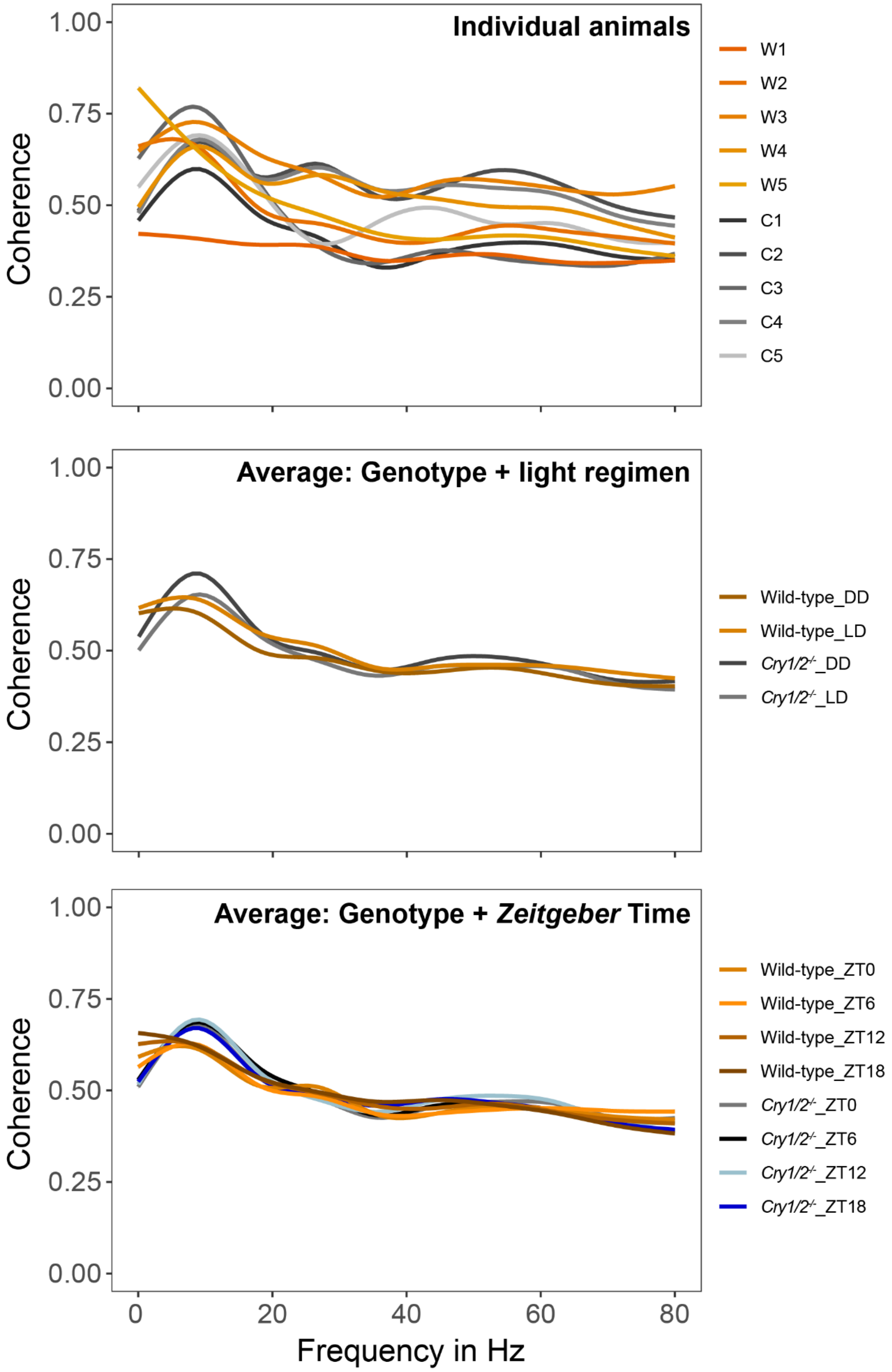

Supplementary Figure 7

Top: Coherence measures, calculated at ZT0, 6, 12, and 18 over 6 minutes of recording, for each individual animal, averaged and smoothed over LD and DD. The plot displays respective smoothed regression lines with 95 % error bands (too small to be visible). Middle: As in A but averaged for all genotype-light regimen combinations. Bottom: As in A but averaged for genotype-light regimen-*Zeitgeber* time combinations.

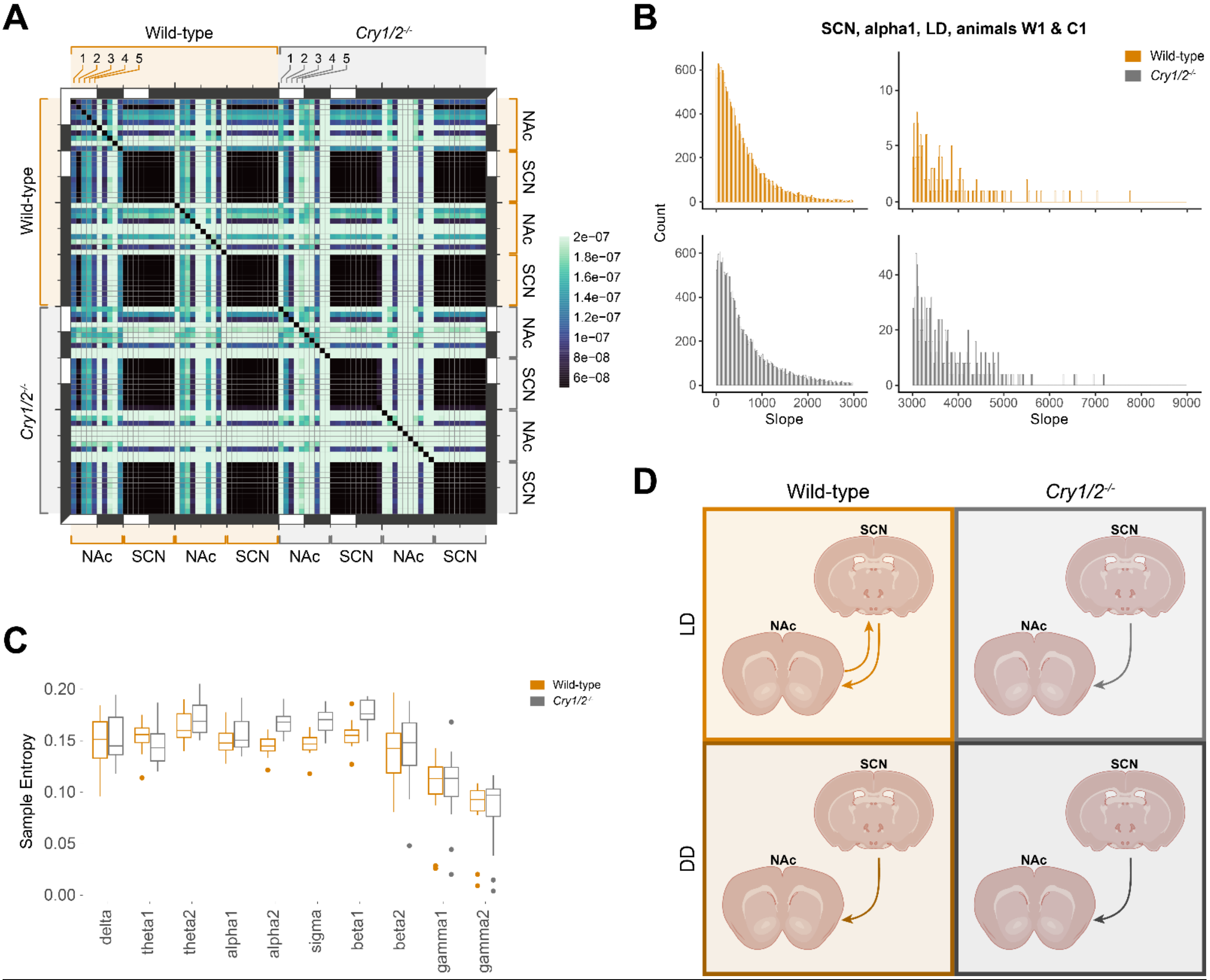

Supplementary Figure 8

(A) Euclidean distance matrix for distances between every combination of every single animal's gamma1 time series in each brain area under each light regimen for each light/dark phase. Dark blue tiles indicate low values, i.e., close Euclidean distance, whereas light green tiles represent high values with larger distances. (B) Histograms of slope parameters of SCN LD alpha1 time series of single representative animals, counting occurrences within a bin width of 20. (C) Sample entropy of individual time series for every frequency, averaged across both brain areas and light regimens, plotted as boxplots with median and whiskers indicating 1.5-fold distance of IQR to the upper/lower quartile. White and black bars in A represent light and dark, respectively, with white-black bars within one brain area block indicating data for LD and black bars for DD. (D) Visual presentation of the results of Granger causality (Table 1) which measures the capability of the time series of one brain region to predict that of another. Brain icons taken from BioRender.com.

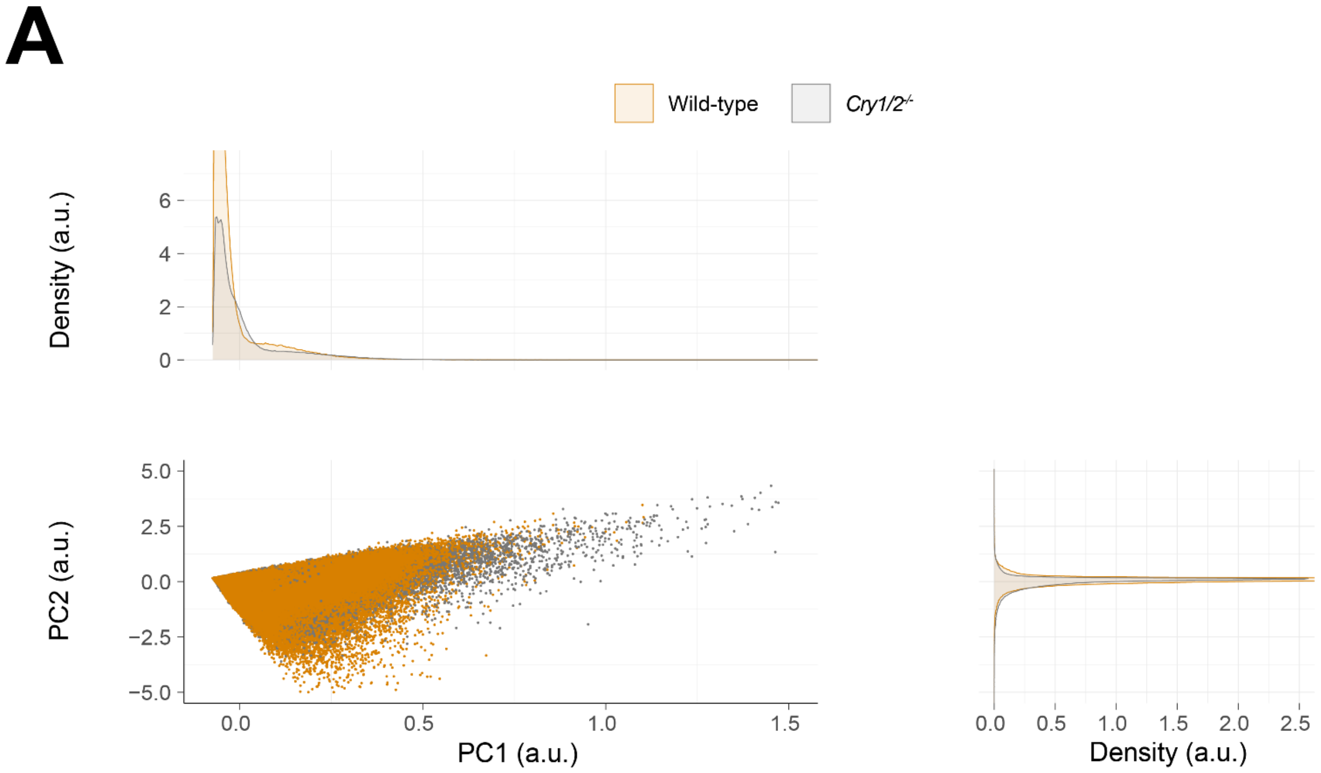

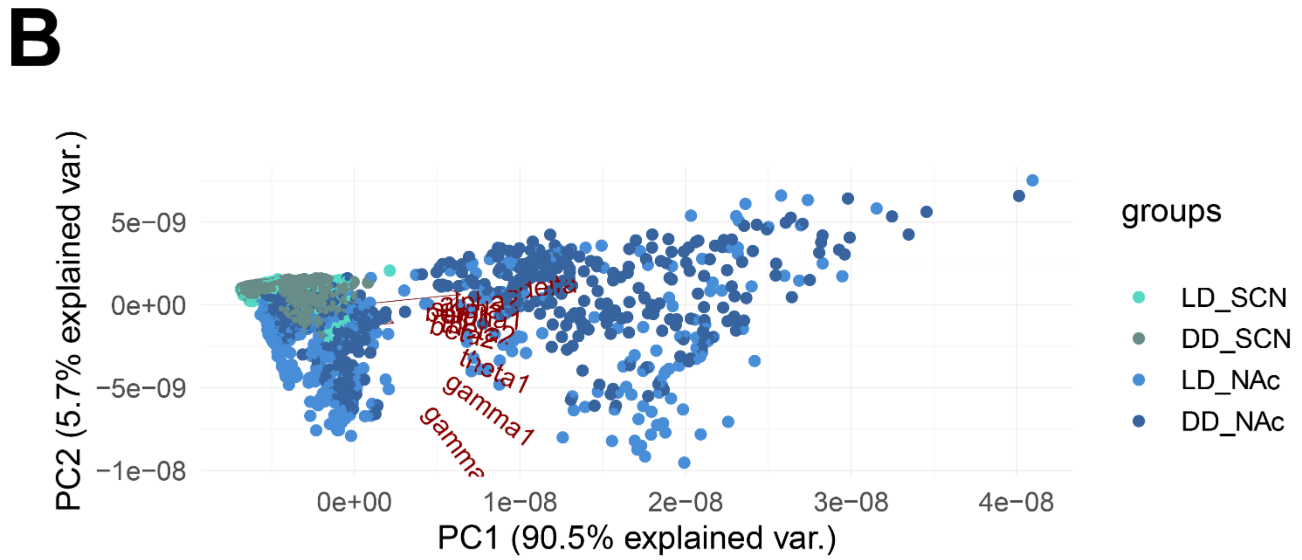

Supplementary Figure 9

(A) PC1 vs PC2 of a PCA of the complete dataset (4-second binned) of all frequencies from all animals under both light regimens from both brain areas with density plots of point clouds. (B) PC1 vs PC2 of a PCA of the complete dataset of the one-hour binned activity of all frequencies from all animals under both light regimens from both brain areas with variable (frequency) arrows. Coloring only distinguishes different light regimen - brain area pairs, but not genotypes. a.u. = arbitrary unit.

Statistics - Type II ANOVA

Figure (Parameter)	Variable	Degrees of Freedom	Sum of Squares	F value	P value of F statistic	Annotation
**1C**	Genotype	1	2178	19.27	1.29E-05	***
**(Block count)**	Light regimen	1	703	6.221	0.0128	*
	Light/Dark phase	1	5714	50.553	2.62E-12	***
	Frequency	9	15994	15.723	< 2E-16	***
	Brain Area	1	146	1.294	0.2557	n.s.
	Genotype:Light regimen	1	192	1.699	0.1927	n.s.
	Genotype:Light/Dark phase	1	173	1.53	0.2164	n.s.
	Genotype:Brain Area	1	34	0.297	0.5856	n.s.
	Residuals	783	88500			
**1E**	Genotype	1	820.2	4.426	0.03889	*
**(AUC LFP)**	Light regimen	1	297.4	1.6048	0.2093	n.s.
	Brain Area	1	10982.8	59.2656	5.60E-11	***
	Light/Dark phase	1	60.8	0.3282	0.56852	n.s.
	Genotype:Light regimen	1	449	2.4229	0.12396	n.s.
	Genotype:Brain Area	1	228.9	1.235	0.27012	n.s.
	Genotype:Light/Dark phase	1	8	0.0432	0.83589	n.s.
	Residuals	72	13342.7			
**2C**	Genotype	1	0.28781	24.3635	4.13E-06	***
**(Sine wave amplitude)**	Light regimen	1	0.31656	26.7966	1.58E-06	***
	Brain Area	1	0.02318	1.9623	0.165	n.s.
	Genotype:Light regimen	1	0.00046	0.0393	0.8433	n.s.
	Genotype:Brain Area	1	0.00645	0.5457	0.4622	n.s.
	Residuals	82	0.96869			
**3C**	Genotype	1	0.1882	4.9835	0.02615	*
**(Correlation value)**	Light regimen	1	0.0025	0.0656	0.79804	n.s.
	Genotype:Light regimen	1	0.0137	0.3616	0.54795	n.s.
	Genotype:Light/Dark phase	2	0.0358	0.4736	0.62311	n.s.
	Residuals	394	14.877			
**4F**	Genotype	1	0.0028622	7.1687	0.0111	*
**(Sample Entropy)**	Light regimen	1	0.0000197	0.0493	0.8255	n.s.
	Genotype:Light regimen	1	0.000115	0.2881	0.5947	n.s.
	Residuals	36	0.0143733			
**4G, S8C**	Genotype	1	0.005838	13.3228	0.0002984	***
**(Sample Entropy)**	Light regimen	1	0.000244	0.556	0.4563246	n.s.
	Brain Area	1	0.015795	36.048	4.45E-09	***
	Frequency	9	0.26044	66.0436	< 2E-16	***
	Genotype:Light regimen	1	0.000043	0.098	0.7543613	n.s.
	Genotype:Brain Area	1	0.003635	8.2957	0.004196	**
	Residuals	385	0.168692			
**S4C**	Genotype	1	352.8	4.4144	0.03914	*
**(Block count)**	Light regimen	1	304.2	3.8063	0.05495	n.s.
	Light/Dark phase	1	156.8	1.962	0.1656	n.s.
	Brain Area	1	76.1	0.9516	0.33258	n.s.
	Genotype:Light regimen	1	281.2	3.5192	0.06472	n.s.
	Genotype:Light/Dark phase	1	22.1	0.2759	0.60101	n.s.
	Genotype:Brain Area	1	16.2	0.2027	0.6539	n.s.
	Residuals	72	5754.2			
**S6G**	Genotype	1	0.20903	220.466	< 2E-16	***
**(Cross-Correlation)**	Light regimen	1	0.00392	4.1345	0.04235	*
	Frequency	9	0	0	1	n.s.
	Genotype:Light regimen	1	0.00002	0.019	0.89032	n.s.
	Residuals	787	0.74617			
**S6G**	Genotype	1	0.7526	624.364	< 2E-16	***
**(Autocorrelation)**	Light regimen	1	0.03197	26.5246	2.93E-07	***
	Brain Area	1	1.53849	1276.35	< 2E-16	***
	Frequency	9	0	0	1	n.s.
	Genotype:Light regimen	1	0.01412	11.7148	0.0006358	***
	Genotype:Brain Area	1	0.00053	0.4416	0.5064413	n.s.
	Residuals	1585	1.91053			
**S6G**	Genotype	1	0.003505	1.144	0.28819	n.s.
**(Cross-Correlation LFP)**	Light regimen	1	0.003417	1.1153	0.29427	n.s.
	Genotype:Light regimen	1	0.015684	5.1197	0.02651	*
	Residuals	76	0.232828			
**S6G**	Genotype	1	0.10223	14.3335	0.0002189	***
**(Autocorrelation LFP)**	Light regimen	1	0.06687	#######	0.0025952	**
	Brain Area	1	0.32248	45.2153	3.25E-10	***
	Genotype:Light regimen	1	0.01374	1.9265	0.1671419	n.s.
	Genotype:Brain Area	1	0.00314	0.4404	0.5079171	n.s.
	Residuals	154	1.09836			
**S7**	Genotype	1	10.1	307.263	< 2E-16	***
**(Coherence measurement)**	Light regimen	1	0.9	26.878	2.17E-07	***
	Zeitgeber time	3	8.4	85.367	< 2E-16	***
	Genotype:Light regimen	1	127.9	3890.51	< 2E-16	***
	Genotype:Zeitgeber time	3	2.6	26.392	< 2E-16	***
	Residuals	921650	30290.9			

Statistics – Linear Sine Fit

Figure (Parameter)	Variable	Estimate	Std. Error	t value	P value of t statistic	Annotation		Adjusted R-squared	Residual Standard Error	Degrees of Freedom	F-Statistic	P value of F statistic
**2A**	**WT LD SCN**							0.2979	0.4539	21596	4584	< 2E-16
**(gamma1 SCN)**	(Intercept)	1.65E-05	3.09E-03	0.005	0.996	n.s.						
	sin(2*pi/24*ZT)	-3.87E-01	4.37E-03	-88.585	< 2E-16	***						
	cos(2*pi/24*ZT)	-1.59E-01	4.37E-03	-36.342	< 2E-16	***						
	**Cry-/- LD SCN**							0.2462	0.4305	21596	3529	< 2E-16
	(Intercept)	-1.53E-06	2.93E-03	-0.001	1	n.s.						
	sin(2*pi/24*ZT)	-2.89E-01	4.14E-03	-69.831	< 2E-16	***						
	cos(2*pi/24*ZT)	-1.94E-01	4.14E-03	-46.704	< 2E-16	***						
	**WT DD SCN**							0.2182	0.4349	21596	3015	< 2E-16
	(Intercept)	-3.83E-07	2.96E-03	0	1	n.s.						
	sin(2*pi/24*ZT)	-2.52E-01	4.19E-03	-60.08	< 2E-16	***						
	cos(2*pi/24*ZT)	-2.06E-01	4.19E-03	-49.18	< 2E-16	***						
	**Cry-/- DD SCN**							0.003502	0.4291	21596	38.96	< 2E-16
	(Intercept)	1.45E-05	2.92E-03	0.005	0.996	n.s.						
	sin(2*pi/24*ZT)	3.54E-02	4.13E-03	8.562	< 2E-16	***						
	cos(2*pi/24*ZT)	-8.87E-03	4.13E-03	-2.147	0.0318	*						
**S5A**	**WT LD NAc**							0.4018	0.4545	21596	7255	< 2E-16
**(gamma1 NAc)**	(Intercept)	2.17E-05	3.09E-03	0.007	0.994	n.s.						
	sin(2*pi/24*ZT)	-4.68E-01	4.37E-03	-106.955	< 2E-16	***						
	cos(2*pi/24*ZT)	-2.42E-01	4.37E-03	-55.408	< 2E-16	***						
	**Cry-/- LD NAc**							0.3141	0.4285	21596	4947	< 2E-16
	(Intercept)	-0.000009	0.002916	-0.003	0.998	n.s.						
	sin(2*pi/24*ZT)	-0.320183	0.004123	-77.651	< 2E-16	***						
	cos(2*pi/24*ZT)	-0.256359	0.004124	-62.169	< 2E-16	***						
	**WT DD NAc**							0.2917	0.4425	21596	4449	< 2E-16
	(Intercept)	-6.65E-06	3.01E-03	-0.002	0.998	n.s.						
	sin(2*pi/24*ZT)	-2.93E-01	4.26E-03	-68.9	< 2E-16	***						
	cos(2*pi/24*ZT)	-2.74E-01	4.26E-03	-64.422	< 2E-16	***						
	**Cry-/- DD NAc**							0.004902	0.4334	21596	54.2	< 2E-16
	(Intercept)	2.24E-05	2.95E-03	0.008	0.993937	n.s.						
	sin(2*pi/24*ZT)	-4.05E-02	4.17E-03	-9.715	< 2E-16	***						
	cos(2*pi/24*ZT)	-1.56E-02	4.17E-03	-3.744	0.000181	***						

Statistics – Student's T-Test

**Figure (Parameter)**	**t value**	**Degrees of Freedom**	**P value of t statistic**	**Annotation**
**2B**	4.338	78.861	4.22E-05	***
**(Sine wave amplitude)**				

Statistics – Generalized Linear Model

Figure (Parameter)	Variable	Degrees of Freedom	Logistic Regression Chi-Square	P value of Chi-Square Statistic	Annotation
**2G**	Genotype	1	28.384	9.95E-08	***
**(Period deviation)**	Light regimen	1	44.788	2.20E-11	***
	Area	1	3.045	0.0810057	n.s.
	Frequency	9	15.625	0.075145	n.s.
	GT:Light regimen	1	3.01	0.0827493	n.s.
	GT:Area	1	11.65	0.0006421	***
**4A-B**	Genotype	1	153.82	< 2E-16	***
**(Euclidean distance)**	Light regimen	1	19.41	1.06E-05	***
	Area	1	463.9	< 2E-16	***
	Light/Dark phase	1	3.69	0.05487	n.s.
	GT:Light regimen	1	38.13	6.61E-10	***
	GT:Light/Dark phase	1	0.36	0.54888	n.s.
**S3A**	Genotype	1	158.4	< 2E-16	***
**(Count of significant oscillatory bouts)**	Light regimen	1	0.3	0.5966	n.s.
	Brain Area	1	159	< 2E-16	***
	Frequency	104	15105.8	< 2E-16	***
	Genotype:Light regimen	1	1	0.3138	n.s.
	Genotype:Brain Area	1	45.7	1.38E-11	***
	Genotype:Frequency	104	867.6	< 2E-16	***
**S3B**	Genotype	1	1508.6	< 2E-16	***
**(Frequency span)**	Light regimen	1	7911.1	< 2E-16	***
	Brain Area	1	7990.8	< 2E-16	***
	Zeitgeber time	3	11558.8	< 2E-16	***
	Genotype:Light regimen	1	979.8	< 2E-16	***
	Genotype:Brain Area	1	1244.5	< 2E-16	***
	Genotype:Zeitgeber time	3	7688.3	< 2E-16	***
**S3D**	Genotype	1	34	6.35E-09	***
**(Event duration)**	Light regimen	1	0	0.5024	n.s.
	Brain Area	1	33	9.77E-09	***
	Frequency	1	46316	< 2E-16	***
	Genotype:Light regimen	1	21	4.54E-06	***
	Genotype:Brain Area	1	0	0.779	n.s.
	Genotype:Frequency	1	132	< 2E-16	***
**S3D**	Genotype	1	5.9	0.01552	*
**(Event interval)**	Light regimen	1	0.1	0.75719	n.s.
	Brain Area	1	6	0.01409	*
	Frequency	1	8168.7	< 2E-16	***
	Genotype:Light regimen	1	3.7	0.05447	n.s.
	Genotype:Brain Area	1	0	0.90957	n.s.
	Genotype:Frequency	1	21.8	2.98E-06	***
**S3E**	Genotype	1	184.23	< 2E-16	***
**(Event power)**	Light regimen	1	2.27	0.132103	n.s.
	Brain Area	1	39.25	3.73E-10	***
	Zeitgeber time	1	831.2	< 2E-16	***
	Genotype:Light regimen	1	10.04	0.001534	**
	Genotype:Brain Area	1	238.8	< 2E-16	***
	Genotype:Zeitgeber time	1	84.24	< 2E-16	***
**S5E**	Genotype	1	6.7366	0.009445	**
**(Period deviation)**	Light regimen	1	1.8594	0.172689	n.s.
	Area	1	7.1897	0.007332	**
	GT:Light regimen	1	0.1167	0.732689	n.s.
	GT:Area	1	0.2544	0.614019	n.s.

Statistics – Pearson Correlation

Figure	Correlation Coefficient R	P value of t statistic	Annotation	Regression Line
3A	WT LD			
(theta1)	0.66	< 2E-16	***	y = 3.5*10-10+0.18x
	Cry-/- LD			
	0.52	< 2E-16	***	y = 7.2*10-10+0.17x
	WT DD			
	0.15	< 2E-16	***	y = 9.9*10-10+0.032x
	Cry-/- DD			
	0.81	< 2E-16	***	y = -4.5*10-10+0.51x
3B	WT LD			
(LFP)	0.62	< 2E-16	***	y = 1.5*10-9+0.2x
	Cry-/- LD			
	0.47	< 2E-16	***	y = 3.5*10-9+0.13x
	WT DD			
	0.098	< 2E-16	***	y = 5.8*10-9+0.018x
	Cry-/- DD			
	0.61	< 2E-16	***	2.2*10-9+0.21x

Statistics – Wilcoxon Rank-Sum Test

**Figure (Parameter)**	**V**	**P value**	**Annotation**
**4D**	710	1.81E-05	***
**Skewness of Slope Histograms**			

Statistics – PCA

Value	PC1	PC2	PC3	PC4	PC5	PC6	PC7	PC8	PC9	PC10
**Proportion of Variance**	0.90480	0.05706	0.02707	0.00732	0.00230	0.00084	0.00051	0.00011	0.00001	0.00000
**Eigenvectors (PCs)**										
**delta**	0.91355307	0.360628698	0.157400622	0.1015002	-0.001515888	0.007275714	0.01030166	0.011209472	-0.001849192	-0.000206078
**theta1**	0.33667236	-0.624576804	-0.642287007	0.1786454	-0.167665042	-0.099639886	0.11800546	-0.008372469	0.007640339	0.003915475
**theta2**	0.11507655	-0.090646491	-0.225986829	-0.2863212	0.521583512	0.14776571	-0.74136145	-0.039915984	0.005599568	-0.019110162
**alpha1**	0.06483569	-0.024091594	-0.130415408	-0.4270821	0.326414899	0.454028861	0.51257988	0.442086543	-0.157556789	0.010816248
**alpha2**	0.03487702	0.004939593	-0.035881166	-0.241364	0.122418943	0.043882344	0.21832549	-0.400963949	0.695753125	0.479366841
**sigma**	0.03882358	-0.004305447	-0.024811593	-0.2574765	0.08161495	-0.002191669	0.21969503	-0.543223425	0.036088004	-0.761734009
**beta1**	0.03146048	-0.010757766	-0.004220663	-0.1975914	0.027665426	-0.075095652	0.09956614	-0.515729927	-0.698686354	0.435173759
**beta2**	0.09211473	-0.091485699	0.101636134	-0.642812	-0.195843968	-0.664092061	-0.04187546	0.277958262	0.033865516	-0.008588483
**gamma1**	0.09813826	-0.298518031	0.290954691	-0.2911492	-0.606521283	0.550585295	-0.23819353	-0.060554418	0.019950382	0.004485906
**gamma2**	0.11312184	-0.611074671	0.630641575	0.1784276	0.405776281	-0.101537153	0.09632548	0.001297851	-0.000665597	-0.001040068

Statistics – Kolmogorov-Smirnov Test

Figure (Parameter)	Difference	P value	Annotation
**5B**	0.15462	< 2E-16	***
**Euclidean Distance PC1 & PC2**			

Statistics – Granger Causality

Direction	Light Regimen	Degrees of Freedom	F value	P value of F statistic	Annotation
		**WT**			
**SCN → NAc**	**LD**	-1	604.9	< 2E-16	***
	**DD**	-1	26.3	0.0001637	***
		**Cry-/-**			
	**LD**	-1	188.49	< 2E-16	***
	**DD**	-1	563.6	< 2E-16	***
**NAc → SCN**		**WT**			
	**LD**	-1	92.05	< 2E-16	***
	**DD**	-1	2.055	0.4216	n.s.
		**Cry-/-**			
	**LD**	-1	3.239	0.3386	n.s.
	**DD**	-1	2.373	0.2793	n.s.
